# New Thiazoline-Tetralin Derivatives and Biological Activity Evaluation

**DOI:** 10.3390/molecules23010135

**Published:** 2018-01-10

**Authors:** Gülhan Turan-Zitouni, Leyla Yurttaş, Aouatef Tabbi, Gülşen Akalın Çiftçi, Halide Edip Temel, Zafer Asım Kaplancıklı

**Affiliations:** 1 Department of Pharmaceutical Chemistry, Faculty of Pharmacy, Anadolu University, 26470 Eskisehir, Turkey; lyurttas@anadolu.edu.tr (L.Y.); zakaplan@anadolu.edu.tr (Z.A.K.); 2 Department of Chemistry, Faculty of Sciences, Mentouri University, 25000 Constantin, Algeria; awateftabbi@yahoo.fr; 3 Department of Biochemistry, Faculty of Pharmacy, Anadolu University, 26470 Eskisehir, Turkey; gakalin@anadolu.edu.tr (G.A.Ç.); heincedal@anadolu.edu.tr (H.E.T.)

**Keywords:** thiazoline, tetralin (tetrahydronaftalene), cytotoxicity, DNA synthesis inhibition, apoptosis, anticholinesterase activity

## Abstract

In this study, novel *N*′-(3-cyclohexyl/phenyl-4-(substituted phenyl)thiazole-2(3*H*)-ylidene)-2-[(5,6,7,8-tetrahydronaphthalen-2-yl)oxy]acetohydrazide (**4a**–**4k**) derivatives were synthesized and their anticancer potency were evaluated on human breast adenocarcinoma cell line (MCF-7), human lung carcinoma cell line (A549) and mouse embryoblast cell line (NIH/3T3) using the MTT method, DNA synthesis inhibition and flow cytometric analysis. Compound **4e** bearing 4-methoxyphenyl moiety exhibited the highest antitumor efficiency against MCF-7 cell line with higher DNA synthesis inhibition and apoptotic cell percentages (ealy+late apoptotic cell). On the other hand, compounds **4f**, **4g**, and **4h** bearing 4-bromo, 4-chloro and 4-florophenyl moieties, respectively caused excellent apoptosis levels against A549 cell line when treated with lower concentration even than cisplatin. Anticholinesterase activity of the compounds were also tested, compound **4h** showed 49.92% inhibition of acetylcholinesterase (AChE).

## 1. Introduction

**Figure 1:**
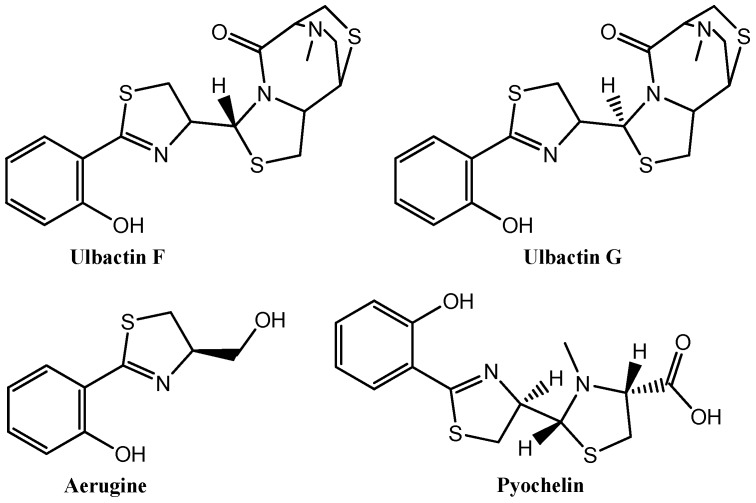
Some thiazoline containing anticancer molecules.

Thiazole ring is an important pharmacologically active heterocylic ring whose mono-, di-, three-substituted, condensed derivatives and reduced analogs (thiazoline, thiazolidine) have been widely studied in medicinal chemistry [1,2,3,4,5,6]. Among these molecules with common origin, thiazoline derivatives have attracted attention due to existing in many biochemical reactions in organisms [7]. Consequently, various thiazoline derivatives were reported with a broad spectrum of activity attributed to this property [8,9]. When considering molecular aspects and biological diversity, a wide variety of thiazoline derivatives were encountered with anticancer activity. From natural sources, oligothiazoline includes some marine products such as tantazole B, mirabazole and thiangazole, and were demonstrated to have high selective cytotoxicity on murine solid tumor [10,11]. Furthermore, bacterial isolates ulbactin F, G, aerugine and pyochelin, which possess tandemly connected thiazoline and thiazolidine ring systems, were declared with tumor cell migration inhibitory activity (Figure 1) [12]. Therewithal, synthetic thiazoline derivatives exhibiting antiproliferative activity were widely studied and well documented in literature [13,14,15,16,17,18,19,20,21,22]. Apoptosis inducing [23] and cell division inhibiting [24] abilities were reported as anticancer activity mechanism.

**Figure 2:**
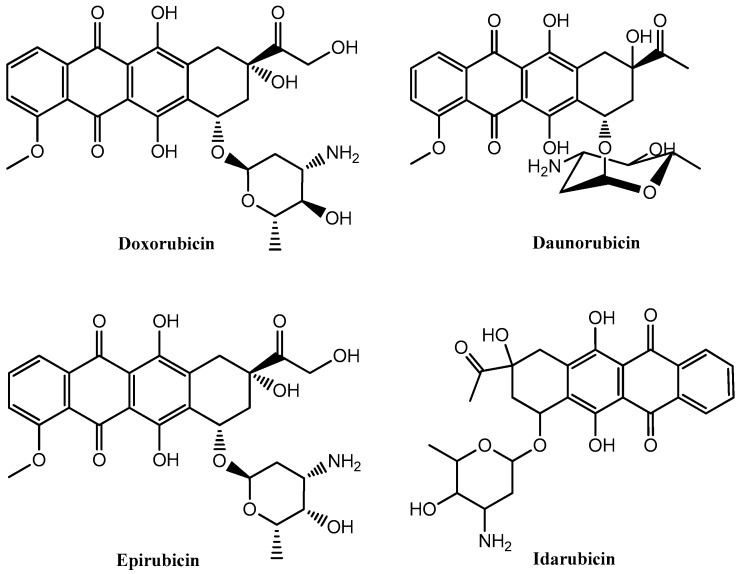
Tetralin including anticancer drugs.

On the other hand, tetrahydronaftalene (tetralin) is an efficient ring, which is found in structures of anthracycline antibiotics doxorubicin, daunorubicin, epirubicin, and idarubicin (Figure 2). These clinically used anticancer drugs have been known to act as DNA intercalators [25]. Another naturally occurring plant-derived podophyllotoxin glycosides have been established with high cytotoxicity since 1950s [26]. Their semisynthetic derivatives etoposide and teniposide are topoisomerase II inhibitor anticancer drugs in clinical use. Due to high toxicity, low water solubility, acquired drug resistance and gastrointestinal discomfort, numerous structural modifications were performed, consequently NK611, NPF, GL-311 and TOP53 molecules have been discovered which are being tested in clinical trials, presently [27,28,29]. Besides, many studies have been carried out to achieve appropriate tetralin derivatives in terms of all aspects to be an efficacious anticancer drug [30,31,32,33,34,35,36,37,38,39,40].

Acetylcholinesterase is an enzyme responsible for the breakdown of cholinergic neurotransmitter acetylcholine. The ability of inhibiting acetylcholinesterase activity is closely associated with myasthenia gravis, Alzheimer, post-operative ileus, bladder distention, glaucoma diseases and Parkinson’s disease dementia [41]. Since the 1980s, regulation of acetylcholinesterase during tumor formation process including adhesion, differentiation, and proliferation stages have been investigated and it was found to show an overexpression of some cholinesterase activities on some tumor varieties. Accordingly, some researches showed that the importance of cholinergic signal system in carcinogenic tissues and reported acetylcholinesterase (AChE) inhibition-dependent reduction of cell proliferation in primary cell lines derived from lung tumor then suggested that main antitumoral effects may be responsible for AChE activity inhibition [42,43].

Considering the above data, we have combined thiazoline and tetralin rings using a hydrazine bridge. We have synthesized novel *N*′-[3-cyclohexyl/phenyl-4-(substituted phenyl)thiazole-2(3*H*)-ylidene]-2-[(5,6,7,8-tetrahydronaphthalen-2-yl)oxy]acetohydrazides (**4a**–**4k**) and evaluate their anticancer effects on MCF-7 and A549 cell lines. The anticholinesterase activity of the compounds was also studied on acetylcholinesterase (AChE) and butyrylcholinesterase (BuChE) enzymes.

## 2. Results and Discussion

### 2.1. Chemistry

**Scheme 1:**
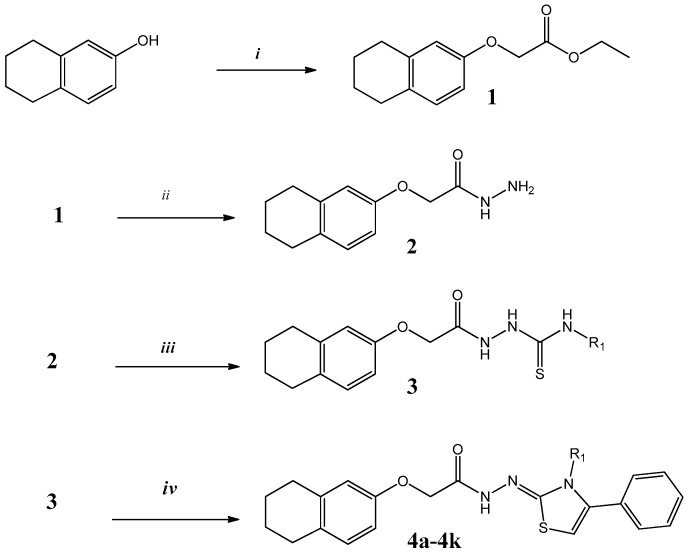
The synthesis of the compounds (**4a**–**4k**). Reactants and reagents: ***i***: ClCH_2_COOEt, 3–5 h, reflux; ***ii***: NH_2_NH_2_·H_2_O, EtOH, 3 h, reflux; ***iii***: C_6_H_5_NCS/C_6_H_11_NCS, EtOH, 2 h, reflux; ***iv***: phenacyl bromide derivative, EtOH, 5 h, reflux.

Eleven new compounds were synthesized in a three step synthetic procedure and this is outlined in Scheme 1. Starting material 5,6,7,8-tetrahydro-2-naphthol was reacted with ethyl 2-chloroethyl acetate to give ethyl 2-[(5,6,7,8-tetrahydronaphthalen-2-yl)oxy]acetate (**1**), this intermediate was hydrazinated with hydrazine hydrate to give ethyl 2-[(5,6,7,8-tetrahydronaphthalen-2-yl)oxy]acetohydrazide (**2**) and these intermediates were reported, previously [44]. The resulting hydrazide was boiled with phenyl and cyclohexyl isothiocyanate in ethanol to give *N*-phenyl/cyclohexyl-2-[2-((5,6,7,8-tetrahydronaphthalen-2-yl)oxy)acetyl]hydrazine carbothioamide (**3a**, **3b**) according to the literature [45]. In the last step these compounds were reacted with phenacyl bromide derivatives to give *N*′-[3-phenyl/cyclohexyl-4-(substituted phenyl)thiazol-2(3*H*)-ylidene]-2-[(5,6,7,8-tetrahydronaphthalen-2-yl)oxy]acetohydrazides (**4a**–**4k**). The substituents were showed in Table 1. The structures of final compounds were confirmed by using spectral and elemental analysis. In the IR spectra of the compounds (see Supplementary materials), characteristic bands belong to N–H and C=O bonds of acetamide moiety were observed at 3109–3199 cm^−1^ and 1712–1745 cm^−1^ whereas the protons of acetamide moiety were observed at 4.75–4.61 ppm as singlets belong to methylene and at 11.79–11.98 ppm belong to amino protons in the ^1^H-NMR spectra of the compounds. –CH_2_ Protons of tetrahydronaftalene ring were detected at 1.67–1.98 ppm and 2.48–2.69 ppm region. In aromatic field, proton of thiazole ring, C_5_–H was seen at about 7.0–7.19 ppm as singlet peak, mostly. In the ^13^C-NMR spectra of the compounds, methylene carbons of acetamide group were resonated at 56.21–66.15 ppm, whereas methylene carbons of tetrahydronaftalene ring were observed at 20.91–31.17 ppm. In the MS, M + 1 peaks were detected for all final compounds. Elemental analysis data was also determined within the accurate range of ±0.4%.

### 2.2. Biological Results

#### 2.2.1. Cytotocity

In order to determine anticancer efficiency of the synthesized compounds, three-step activity studies were carried out. Accordingly, cytotoxicity and DNA synthesis inhibition experiments and tests to determine apoptosis/necrosis ratios were performed. Tested tumor cells were human breast adenocarcinoma cell line (MCF-7), human lung carcinoma cell line (A549) and healthy cell mouse embryoblast cell line (NIH/3T3) were used to detect cytotoxicity of the compounds according to classical MTT method. The calculated inhibitory concentrations (IC_50_) and selectivity index (SI) are represented in Table 2.

Compounds **4b** (IC_50_ = 69.2 μM) and **4d** (IC_50_ = 71.8 μM) showed the highest cytotoxicity against MCF-7 cell line. However, compounds **4e** and **4k** also exhibited considerable cytotoxic activity. None of these compounds were found as active as standard drug cisplatin. Among these four compounds **4b** and **4k** showed toxicity to normal cells. When we compare, antiproliferative effects of the compounds, their potency against A549 was found to be greater than against MCF-7 cells. It has been determined that compounds **4f**, **4g** and **4h** act at a lower concentration than the inhibition concentration of cisplatin which possessed 4-bromo, 4-chloro and 4-fluoro substituent moieties respectively on A549 cell line. Besides, these compounds exhibited selectivity due to their low cytotoxicity on healthy cells with selectivity index in between 2.7 and 7.8. However, the IC_50_ values of compounds **4c**, **4d**, **4i** and **4k** were not determined at the highest concentration tested against A549. Additionally, compounds **4a**, **4b** and **4j** exhibited moderate antiproliferative activity.

#### 2.2.2. DNA Synthesis Inhibition

**Figure 3:**
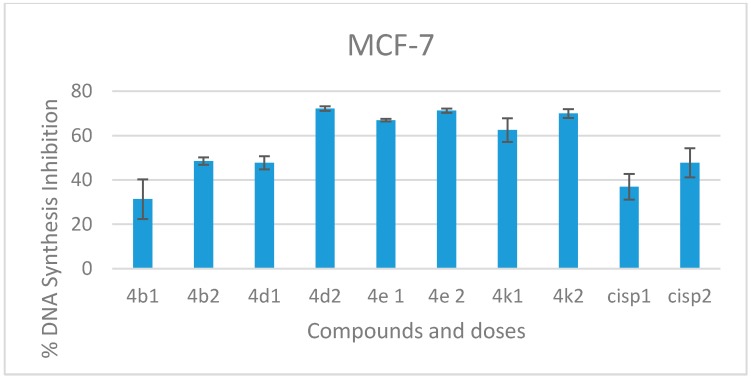
DNA synthesis inhibitory activity of compounds **4b**, **4d**, **4e**, **4k** and cisplatin on MCF-7 cells. Mean percent absorbance of untreated control cells were assumed 0%. In addition, two different concentrations (1: IC_50_/2; 2: IC_50_ µM) of test compounds and cisplatin were given. Data points represent means for three independent wells ± SD. *p* < 0.05.

DNA synthesis inhibition study, which was considered as the second stage in anticancer activity studies, have been conducted on compounds with high cytotoxicity. Compounds **4b**, **4d**, **4e**, and **4k** were determined to possess more cytotoxic activity on the MCF cell line. The graph generated according to the percent inhibitions of the selected compounds at a: IC_50_/2 and b: IC_50_ concentrations is shown in Figure 3. Compound **4b** inhibited DNA synthesis with ratio of 31.39% at 34.6 μM and 48.50% at 69.2 μM concentration on MCF-7 cell line. It was seen this ratio was very close to the inhibition exhibited by cisplatin. The inhibition rates for compounds **4d**, **4e** and **4k** were calculated higher than the rate of standard, ranging from 47.75% to 72.15%. Among these three, compound **4d** exhibited the best inhibition potential of 47.75% at 36.9 μM concentration and 72.15% at 71.8 μM on MCF-7 cells.

**Figure 4:**
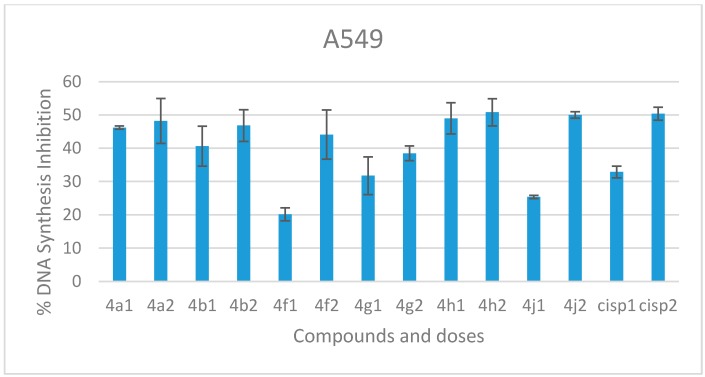
DNA synthesis inhibitory activity of compounds **4a**, **4b**, **4f**, **4g**, **4h**, **4j** and cisplatin on A549 cells. Mean percent absorbance of untreated control cells were assumed 0%. In addition, two different concentrations (1: IC_50_/2; 2: IC_50_ µM) of test compounds and cisplatin were given. Data points represent means for three independent wells ± SD. *p* < 0.05.

DNA synthesis inhibition studies have also been conducted on those compounds that exhibit high cytotoxic activity on A549 cells. Compounds **4a**, **4b**, **4f**, **4g** and **4j** were selected for this study. The graph based on the obtained results is shown in Figure 4. DNA synthesis inhibition on A549 revealed that these six compounds showed close inhibition values of 20.13–50.33%. The compound **4h** bearing 4-fluoro substitution on phenyl ring showed the highest inhibitory effect. The inhibition percentages were specified as 8.97% at 24.3 μM and 50.78% at 48.6 μM whereas these values were established 32.86% at 54.2 μM and 50.33% at 108.3 μM for standard drug cisplatin.

#### 2.2.3. Apoptosis/Necrosis Detection

**Figure 5:**
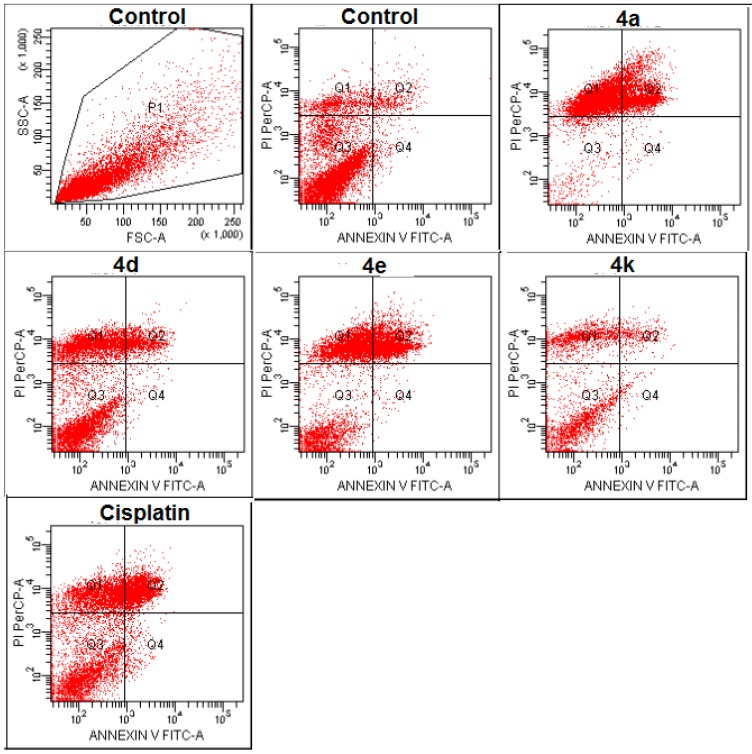
Flow cytometric diagrams of MCF-7 cells treated with compounds **4a**, **4d**, **4e**, **4k**, and cisplatin.

**Figure 6:**
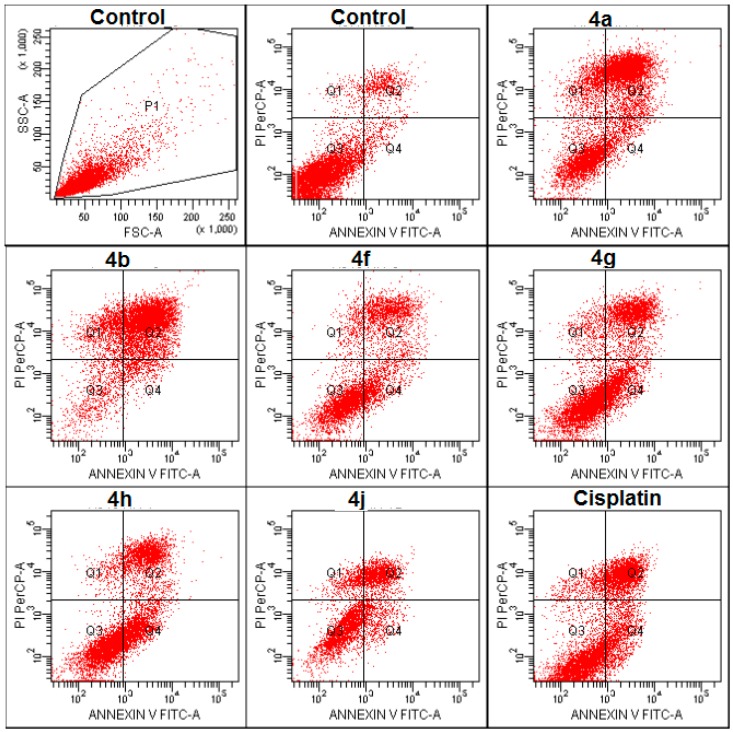
Flow cytometric diagrams of A549 cells treated with compounds **4a**, **4b**, **4f**, **4g**, **4h**, **4j** and cisplatin.

To determine the death mechanism of the most active compounds, flow cytometric analysis was done according to the instructions of the Annexin V-FITC Apoptosis Detection Kit. The results were calculated in percentage of which the ratios were demonstrated as early apoptotic, late apoptotic, viable and necrotic cells. The numerical values were represented in Table 3 and diagrams in Figure 5 and Figure 6. Compounds **4b**, **4d**, **4e** and **4k** against MCF-7 cell line and compounds **4a**, **4b**, **4f**, **4g**, **4h** and **4j** against A549 cells were tested in flow cytometry at IC_50_ concentrations of the compounds. Against MCF-7 cell line, compounds **4b** and **4e** exhibited 29.8% and 32.5% apoptosis (ealy + late apoptotic cell percentage), respectively whereas cisplatin caused 29.9% apoptosis of the cells. Other two compounds did not showed prominent activity. All selected compounds for flow cytometric analysis on A549 cell line displayed marvelous activity that apoptotic cell ratios were found even higher than cisplatin caused (40.2%). Among them, compound **4b** showed the highest apoptosis level of 73.6% which was a great value for such a study. Besides, compounds **4a** and **4h** caused apoptosis over 50% and compounds **4f**, **4g** and **4j** caused apoptosis over 41.5%. Among these compounds, the results of compounds **4f**, **4g** and **4h** bearing *p*-halogenated phenyl moiety are more meaningful, which, is because they have lower IC_50_ values than cisplatin. Consequently, the dose administered to this test is lesser, but apoptotic cell percentage found is higher. In addition, these three compounds have exhibited non-toxic profile which is a required property for druglikeness of a molecule.

#### 2.2.4. Anticholinesterase Activity

The inhibitory effects of the compounds on the acetylcholinesterase (AChE) and butyrylcholinesterase (BuChE) enzymes were investigated. The efficacy of the compounds is compared to the anticholinesterase effective donepezil drug molecule used in Alzheimer's treatment. The results obtained are shown in Table 4.

Based on the enzyme inhibition studies, AChE and BuChE inhibitions were determined at 80 μg/mL (~160 µM) concentrations of the compounds. Compound **4h** showed the highest acetylcholinesterase inhibition with 49.92%. It was followed by compounds **4j** with 44.96%, **4d** with 43.22%, **4f** with 42.87% and **4i** with 41.85%. Compounds **4b** and **4e** exhibited the lowest inhibition among remaining others. Since the compounds did not provide over 50% enzyme inhibition, they were not studied at lower doses to detect inhibition concentrations (IC_50_). The IC_50_ value of the standard drug, donepezil, was determined to be (11.8 ± 0.7) × 10^−3^ μM. The inhibition of butyrylcholinesterase enzyme at concentration of 80 μg/mL was found in between 15.89% and 33.19% for compounds **4a**, **4b**, **4c** and **4d** while other compounds did not inhibit this enzyme. Compounds **4f**, **4g**, **4h** and **4j** are the most active compounds on A549 cancer cells. The AChE inhibition effect of these substances may enhance antiproliferative activity of them.

## 3. Materials and Methods

### 3.1. Chemistry

All chemicals were purchased from Sigma-Aldrich Chemical Co., (Sigma-Aldrich Corp., St. Louis, MO, USA) and Merck (Merck, Darmstadt, Germany). All melting points (m.p.) were determined by Electrothermal 9300 digital melting point apparatus (Electrothermal, Essex, UK) and are uncorrected. All the reactions were monitored by thin-layer chromatography (TLC) using Silica gel 60 F254 TLC plates (Merck, Darmstadt, Germany). Spectroscopic data were recorded with the following instruments: IR, Shimadzu 8400S spectrophotometer (Shimadzu, Tokyo, Japan); ^1^H-NMR, Agilent 400 MHz NMR spectrometer (Agilent Technologies, Palo Alto, CA, USA); ^13^C-NMR, Agilent 100 MHz NMR spectrometer (Agilent Technologies, Palo Alto, CA, USA); Shimadzu 8040 LC/MS/MS system (Shimadzu, Tokyo, Japan), and elemental analyses were performed on a Perkin Elmer EAL 240 elemental analyser (Perkin Elmer, Norwalk, CT, USA).

*General procedure for the synthesis of N′-[3-cyclohexyl/phenyl-4-(substituted phenyl)thiazole-2(3H)-ylidene]-2-[(5,6,7,8-tetrahydronaphthalen-2-yl)oxy]acetohydrazide* (**4a**–**4k**). The synthesized intermediates *N*-phenyl/cyclohexyl-2-[2-((5,6,7,8-tetrahydronaphthalen-2-yl)oxy)acetyl]hydrazine carbothioamide (**3a**, **3b**) were refluxed with phenacyl bromide derivatives for 5 h in ethanol. After overnight standing in a cool place, crystals of final thiazole compounds were filtered off.

*N′-(3-Cyclohexyl-4-phenylthiazole-2(3H)-ylidene)-2-[(5,6,7,8-tetrahydronaphthalen-2-yl)oxy]acetohydrazide* (**4a**): Yield: % 68. m.p. 277 °C. IR ν_max_ (cm^−1^): 3199 (N–H), 3070 (aromatic C–H), 2854 (aliphatic C–H), 1745 (amide C=O), 1595–1442 (C=C and C=N), 1215–1004 (C–N and C–O). ^1^H-NMR (400 MHz, DMSO-*d*_6_, ppm): δ 1.13–2.60 (19H, m, CH_2_), 4.61 (2H, s, OCH_2_), 6.46–7.51 (9H, m, Ar–H), 11.85 (1H, s, NH). ^13^C-NMR (100 MHz, DMSO-*d*_6_, ppm) δ 22.5, 22.8, 24.3, 24.5, 27.9, 28.9, 31.2, 55.2, 58.5, 66.0, 100.9, 112.1, 114.1, 114.3, 118.9, 129.5, 129.5, 130.1, 137.6, 140.8, 155.1, 160.6, 167.3. HRMS (*m*/*z*): [M + H]^+^ for C_27_H_31_N_3_O_2_S calculated: 461.62; found: 462.2195. For C_27_H_31_N_3_O_2_S calculated: 70.25% C, 6.77% H, 9.10% N; found: 70.23% C, 6.76% H, 9.12% N.

*N′-[3-Cyclohexyl-4-(4-methoxyphenyl)thiazole-2(3H)-ylidene]-2-[(5,6,7,8-tetrahydronaphthalen-2-yl)oxy]acetohydrazide* (**4b**): Yield: % 71. m.p. 266 °C. IR ν_max_ (cm^−1^): 3182 (N–H), 3073 (aromatic C–H), 2972 (aliphatic C–H), 1714 (amide C=O), 1602–1502 (C=C and C=N), 1247–1037 (C–N and C–O). ^1^H-NMR (400 MHz, DMSO-*d*_6_, ppm): δ 1.12–2.61 (19H, m, CH_2_), 4.62 (2H, s, OCH_2_), 6.45–6.49 (2H, m, Ar–H), 6.85 (1H, d, *J* = 8.4 Hz, Ar–H), 6.95 (2H, d, *J* = 8.8 Hz), 7.12 (1H, s, Ar–H), 7.43 (2H, d, *J* = 8.8 Hz, Ar–H), 11.79 (1H, s, NH). ^13^C-NMR (100 MHz, DMSO-*d*_6_, ppm) δ 22.5, 22.7, 27.8, 28.9, 66.1, 112.0, 114.3, 122.6, 128.1, 129.4, 129.5, 129.9, 130.0, 130.7, 131.4, 132.6, 137.5, 137.7, 155.1, 167.8. HRMS (*m*/*z*): [M + H]^+^ for C_28_H_33_N_3_O_3_S calculated: 491.65; found: 492.2296. For C_28_H_33_N_3_O_3_S calculated: 68.40% C, 6.77% H, 8.55% N; found: 68.42% C, 6.76% H, 8.56% N.

*N′-[3,4-Diphenylthiazole-2(3H)-ylidene]-2-[(5,6,7,8-tetrahydronaphthalen-2-yl)oxy]acetohydrazide* (**4c**): Yield: % 73. m.p. 253 °C. IR ν_max_ (cm^−1^): 3120 (N–H), 3053 (aromatic C–H), 2987 (aliphatic C–H), 1714 (amide C=O), 1600–1444 (C=C and C=N), 1217–1055 (C–N and C–O). ^1^H-NMR (400 MHz, DMSO-*d*_6_, ppm): δ 1.98 (4H, br, CH_2_), 2.56–2.61 (4H, m, CH_2_), 4.65 (2H, s, OCH_2_), 6.49–7.58 (13H, m, Ar–H), 7.07 (1H, s, thiazole–H), 11.98 (1H, br, NH). ^13^C-NMR (100 MHz, DMSO-*d*_6_, ppm) δ 22.6, 22.8, 22.9, 24.5, 25.2, 27.9, 29.0, 30.7, 56.2, 60.2, 112.6, 112.6, 114.5, 123.8, 129.6, 129.8, 129.8, 137.7, 140.2, 149.1, 150.1, 151.7, 155.1, 192.6. HRMS (*m*/*z*): [M + H]^+^ for C_27_H_25_N_3_O_2_S calculated: 455.58; found: 456.1719. For C_27_H_25_N_3_O_2_S calculated: 71.18% C, 5.53% H, 9.22% N; found: 71.20% C, 5.54% H, 9.23% N.

*N′-[3-Phenyl-4-(4-methylphenyl)thiazole-2(3H)-ylidene]-2-[(5,6,7,8-tetrahydronaphthalen-2-yl)oxy]acetohydrazide* (**4d**): Yield: % 72. m.p. 258 °C. IR ν_max_ (cm^−1^): 3109 (N–H), 3047 (aromatic C–H), 2860 (aliphatic C–H), 1745 (amide C=O), 1599–1496 (C=C and C=N), 1199–1074 (C–N and C–O). ^1^H-NMR (400 MHz, DMSO-*d*_6_, ppm): δ 1.67 (4H, br, CH_2_), 2.32 (3H, s, CH_3_), 2.56–2.59 (4H, m, CH_2_), 4.65 (2H, s, OCH_2_), 6.48–7.56 (12H, m, Ar–H), 7.00 (1H, s, thiazole–H), 11.98 (1H, br, NH). ^13^C-NMR (100 MHz, DMSO-*d*_6_, ppm) δ 22.4, 22.7, 27.8, 28.9, 66.0, 112.1, 114.1, 122.7, 123.6, 129.3, 129.4, 129.5, 130.1, 133.7, 137.4, 138.1, 147.6, 155.1, 168.0. HRMS (*m*/*z*): [M + H]^+^ for C_28_H_27_N_3_O_2_S calculated: 469.60; found: 470.1885. For C_28_H_27_N_3_O_2_S calculated: 71.62% C, 5.80% H, 8.95% N; found: 71.61% C, 5.79% H, 8.94% N.

*N′-[3-Phenyl-4-(4-methoxyphenyl)thiazole-2(3H)-ylidene]-2-[(5,6,7,8-tetrahydro naphthalen-2-yl)oxy]acetohydrazide* (**4e**): Yield: % 67. m.p. 240 °C. IR ν_max_ (cm^−1^): 3109 (N–H), 3047 (aromatic C–H), 2860 (aliphatic C–H), 1745 (amide C=O), 1599–1496 (C=C and C=N), 1199–1074 (C–N and C–O). ^1^H-NMR (400 MHz, DMSO-*d*_6_, ppm): δ 1.68 (4H, br, CH_2_), 2.48–2.59 (4H, m, CH_2_), 3.77 (3H, s, OCH_3_), 4.65 (2H, s, OCH_2_), 6.47–7.57 (13H, m, Ar–H), 11.98 (1H, br, NH). ^13^C-NMR (100 MHz, DMSO-*d*_6_, ppm) δ 22.6, 22.9, 27.9, 29.0, 66.0, 112.2, 114.4, 123.5, 127.3, 128.4, 128.7, 129.5, 129.7, 129.7, 131.1, 130.3, 137.6, 140.6, 155.2, 167.7. HRMS (*m*/*z*): [M + H]^+^ for C_28_H_27_N_3_O_3_S calculated: 485.60; found: 486.1834. For C_28_H_27_N_3_O_3_S calculated: 69.26% C, 5.60% H, 8.65% N; found: 69.24% C, 5.61% H, 8.66% N.

*N′-[3-Phenyl-4-(4-bromophenyl)thiazole-2(3H)-ylidene]-2-[(5,6,7,8-tetrahydronaphthalen-2-yl)oxy]acetohydrazide* (**4f**): Yield: % 74. m.p. 272 °C. IR ν_max_ (cm^−1^): 3122 (N–H), 3039 (aromatic C–H), 2935 (aliphatic C–H), 1712 (amide C=O), 1598–1454 (C=C and C=N), 1313–1006 (C–N and C–O). ^1^H-NMR (400 MHz, DMSO-*d*_6_, ppm): δ 1.67 (4H, br, CH_2_), 2.50–2.63 (4H, m, CH_2_), 4.67 (2H, d, *J* = 4.4 Hz, OCH_2_), 6.48–6.51 (2H, m, Ar–H), 6.87 (1H, d, *J* =7.6 Hz, Ar–H), 7.04 (1H, s, thiazole–H), 7.33–7.38 (3H, m, Ar–H), 7.45 (2H, d, *J* = 8.4 Hz), 7.52–7.56 (2H, m, Ar–H), 7.60 (2H, d, *J* = 8.4 Hz, Ar–H), 11.89 (1H, br, NH). ^13^C-NMR (100 MHz, DMSO-*d*_6_, ppm) δ 22.6, 22.9, 28.0, 29.0, 66.0, 112.2, 114.4, 123.2, 126.3, 128.8, 129.5, 129.6, 130.1, 130.3, 134.9, 137.6, 139.26, 155.2, 167.9. HRMS (*m*/*z*): [M + H]^+^ for C_27_H_24_N_3_O_2_SBr calculated: 534.47; found: 536.0798. For C_27_H_24_N_3_O_2_SBr calculated: 60.68% C, 4.53% H, 7.86% N; found: 60.67% C, 4.52% H, 7.87% N.

*N′-[3-Phenyl-4-(4-chlorophenyl)thiazole-2(3H)-ylidene]-2-[(5,6,7,8-tetrahydronaphthalen-2-yl)oxy]acetohydrazide* (**4g**): Yield: % 67. m.p. 268 °C. IR ν_max_ (cm^−1^): 3122 (N–H), 3032 (aromatic C–H), 2972 (aliphatic C–H), 1712 (amide C=O), 1593–1456 (C=C and C=N), 1313–1055 (C–N and C–O). ^1^H-NMR (400 MHz, DMSO-*d*_6_, ppm): δ 1.68 (4H, br, CH_2_), 2.50–2.62 (4H, m, CH_2_), 4.64 and 4.70 (2H, d, *J* = 15.6 Hz, OCH_2_), 6.49–6.51 (2H, m, Ar–H), 6.87 (1H, d, *J* = 8.8 Hz, Ar–H), 7.05 (1H, s, thiazole–H), 7.35–7.56 (9H, m, Ar–H), 11.93 (1H, br, NH). ^13^C-NMR (100 MHz, DMSO-*d*_6_, ppm) δ 20.9, 22.6, 22.9, 29.0, 66.0, 112.2, 114.4, 123.5, 124.4, 128.8, 129.3, 129.5, 129.6, 130.4, 137.6, 139.9, 140.7, 155.2, 167.7. HRMS (*m*/*z*): [M + H]^+^ for C_27_H_24_N_3_O_2_SCl calculated: 490.02; found: 490.1328. For C_27_H_24_N_3_O_2_SCl calculated: 66.18% C, 4.94% H, 7.58% N; found: 66.16% C, 4.93% H, 8.57% N.

*N′-[3-Phenyl-4-(4-florophenyl)thiazole-2(3H)-ylidene]-2-[(5,6,7,8-tetrahydronaphthalen-2-yl)oxy]acetohydrazide* (**4h**): Yield: % 72. m.p. 257 °C. IR ν_max_ (cm^−1^): 3116 (N–H), 3043 (aromatic C–H), 2916 (aliphatic C–H), 1714 (amide C=O), 1600–1494 (C=C and C=N), 1313–1035 (C–N and C–O). ^1^H-NMR (400 MHz, DMSO-*d*_6_, ppm): δ 1.67 (4H, br, CH_2_), 2.57–2.62 (4H, m, CH_2_), 4.62 and 4.69 (2H, dd, *J* = 15.6 Hz, OCH_2_), 6.50–6.53 ( 2H, m, Ar–H), 6.86 (1H, d, *J* = 8.0 Hz, Ar–H), 7.03 (1H, s, thiazole–H), 7.21–7.56 (9H, m, Ar–H), 11.93 (1H, br, NH). ^13^C-NMR (100 MHz, DMSO-*d*_6_, ppm) δ 22.6, 22.8, 27.9, 29.0, 66.0, 112.1, 114.4, 123.1, 123.6, 126.6, 129.5, 129.6, 130.2, 130.2, 131.6, 137.6, 139.3, 155.2, 167.8. HRMS (*m*/*z*): [M + H]^+^ for C_27_H_24_FN_3_O_2_S calculated: 473.57; found: 474.1629. For C_27_H_24_FN_3_O_2_S calculated: 68.48% C, 5.11% H, 8.87% N; found: 68.49% C, 5.12% H, 8.88% N.

*N′-[3-Phenyl-4-(3-nitrophenyl)thiazole-2(3H)-ylidene]-2-[(5,6,7,8-tetrahydronaphthalen-2-yl)oxy]acetohydrazide* (**4i**): Yield: % 69. m.p. 252 °C. IR ν_max_ (cm^−1^): 3118 (N–H), 3045 (aromatic C–H), 2949 (aliphatic C–H), 1710 (amide C=O), 1604–1344 (C=C and C=N), 1217–1062 (C–N and C–O). ^1^H-NMR (400 MHz, DMSO-*d*_6_, ppm): δ 1.64 (4H, br, CH_2_), 2.47–2.57 (4H, m, CH_2_), 4.52 and 6.72 (2H, dd, *J* = 15.6 Hz, OCH_2_), 6.41–6.43 (2H, m, Ar–H), 6.74 (1H, d, *J* = 8.0 Hz, Ar–H), 7.16 (1H, s, thiazole–H), 7.30–8.29 (9H, m, Ar–H), 11.95 (1H, br, NH). ^13^C-NMR (100 MHz, DMSO-*d*_6_, ppm) δ 22.6, 22.8, 27.9, 29.0, 55.3, 66.1, 112.2, 114.1, 114.4, 119.4, 123.6, 129.5, 129.6, 130.0, 130.6, 137.6, 140.6, 155.2, 160.5, 167.7. HRMS (*m*/*z*): [M + H]^+^ for C_27_H_24_N_4_O_4_S calculated: 500.57; found: 501.1567. For C_27_H_24_N_4_O_4_S calculated: 64.79% C, 4.83% H, 11.19% N; found: 64.78% C, 4.82% H, 11.18% N.

*N′-[3-Phenyl-4-(4-nitrophenyl)thiazole-2(3H)-ylidene]-2-[(5,6,7,8-tetrahydronaphthalen-2-yl)oxy]acetohydrazide* (**4j**): Yield: % 67. m.p. 268 °C. IR ν_max_ (cm^−1^): 3126 (N–H), 3037 (aromatic C–H), 2931 (aliphatic C–H), 1714 (amide C=O), 1602–1338 (C=C and C=N), 1288–1035 (C–N and C–O). ^1^H-NMR (400 MHz, DMSO-*d*_6_, ppm): δ 1.65 (4H, br, CH_2_), 2.50–2.57 (4H, m, CH_2_), 4.61 and 4.75 (2H, dd, *J* = 15.6 Hz, OCH_2_), 6.48–6.54 ( 2H, m, Ar–H), 6.81 (1H, d, *J* = 8.0 Hz, Ar–H), 7.19 (1H, s, thiazole–H), 7.30–8.20 (9H, m, Ar–H), 11.94 (1H, br, NH). ^13^C-NMR (100 MHz, DMSO-*d*_6_, ppm) δ 22.6, 22.8, 27.9, 29.0, 66.03, 112.2, 114.3, 115.7, 115.9, 123.3, 123.8, 127.4, 129.6, 129.6, 130.3, 130.8, 130.9, 137.6, 139.5, 155.1, 161.7, 164.2, 167.8. HRMS (*m*/*z*): [M + H]^+^ C_27_H_24_N_4_O_4_S için calculated: 500.57; found: 501.1572. For C_27_H_24_N_4_O_4_S calculated: 64.79% C, 4.83% H, 11.19% N; found: 64.80% C, 4.84% H, 11.20% N.

*N′-[3-Phenyl-4-(2,3-dichlorophenyl)thiazole-2(3H)-ylidene]-2-[(5,6,7,8-tetrahydronaphthalen-2-yl)oxy]acetohydrazide* (**4k**): Yield: % 75. m.p. 272 °C. IR ν_max_ (cm^−1^): 3109 (N–H), 3047 (aromatic C–H), 2916 (aliphatic C–H), 1732 (amide C=O), 1610–1305 (C=C and C=N), 1219–1028 (C–N and C–O). ^1^H-NMR (400 MHz, DMSO-*d*_6_, ppm): δ 1.68 (4H, br, CH_2_), 2.49–2.62 (4H, m, CH_2_), 4.63 and 4.72 (2H, dd, *J* = 15.6 Hz, OCH_2_), 6.48–6.50 (2H, m, Ar–H), 6.84 (1H, d, *J* = 8.0 Hz, Ar–H), 7.04 (1H, s, thiazole–H), 7.27–7.82 (8H, m, Ar–H), 11.92 (1H, br, NH). ^13^C-NMR (100 MHz, DMSO-*d*_6_, ppm) δ 22.4, 22.7, 27.8, 28.8, 66.2, 111.9, 114.1, 122.9, 124.4, 128.8, 129.4, 129.5, 130.6, 130.2, 134.7, 137.4, 138.0, 147.4, 155.0, 168.0. HRMS (*m*/*z*): [M + H]^+^ for C_27_H_23_Cl_2_N_3_O_2_S calculated: 524.46; found: 524.0938. For C_27_H_23_Cl_2_N_3_O_2_S calculated: 61.83% C, 4.42% H, 8.01% N; found: 61.82% C, 4.43% H, 8.02%N.

### 3.2. Biochemistry

#### 3.2.1. MTT Assay (Mitochondrial Activity)

This is a colorimetric assay that measures the reduction of 3-(4,5-dimethylthiazol-2-yl)-2,4,diphenyltetrazolium bromide (MTT) by mitochondria [46]. All final compounds were tested against human breast adenocarcinoma cell line (MCF-7), human lung carcinoma cell line (A549) and mouse embryooblast cell line (NIH/3T3) to determine their cytotoxic profile. Cancer cells were cultured in RPMI medium (Hyclone, Thermo Scientific, Waltham, MA, USA) at 37 °C in a humidified atmosphere of 95% air and 5% CO_2_. The studied cells were situated at 2 × 10^4^ cells into each well of 96-well microtiter tissue culture plates (Nunc, Denmark) and incubated for 24 h. After that, test compounds were dissolved in DMSO and added to culture wells at varying concentrations (85–1000 µM). After a 24 h incubating period, 20 mL MTT solution (5 mg/mL MTT powder in PBS) was added to each well and the cells were incubated for 4 h at 37 °C. The purple formazan crystals produced were dissolved in DMSO and the absorbance was read by ELISA reader (OD570 nm). The percent values of cell proliferations were calculated relative to controls, whose cell proliferations were accepted as 100% [47].

#### 3.2.2. DNA Synthesis Inhibition

The 5-bromo-2′-deoxy-uridine (BrdU) cell proliferation assay [48] was used to detect the proliferation of A549 and C6 cells. This method was performed in the 96-well flat-bottomed microtiter plates by using cisplatin as positive control drug. A549 and C6 cells were collected from cell cultures by 0.25% trypsin/EDTA solution and counted in a hemocytometer. Suspensions of cell lines were seeded into 96-well flat-bottomed microtiter plates at a density of 1 × 10^3^ cells/mL. The tumor cell lines were cultured in the presence of various doses of the test compounds or standard drugs. Microtiter plates were incubated at 37 °C in a 5% CO_2_/95% air humidified atmosphere for 24 h and 48 h. At the end of each day, the cells were labelled with 10 mL BrdU solution for 2 h and then fixed. Anti-BrdU-POD (100 mL) was added and incubated for 90 min. Finally, microtiter plates were washed with phosphate buffered saline (PBS) three times and the cells were incubated with substrate solution until the color was sufficient for photometric detection. Absorbance of the samples was measured with an ELx808-IUBio-Tek apparatus at 492 nm. The growth percentage was evaluated spectrophotometrically versus untreated controls with used cell viability of growth assay. Results for each spectrophotometrical measure were noticed as percent of growth inhibition. All experiments were done in triplicates.

#### 3.2.3. Flow Cytometric Analysis

Evaluation of apoptosis was carried out through the Annexin V-FITC Apoptosis Detection Kit from BD, Pharmingen, according to the manufacturer’s instruction. This assay was performed on C6 and A549 cell lines with selected compounds which crossed the second step of anticancer activity. After induced apoptosis of cancer cells by the addition of IC_50_ concentrations of selected compounds and positive controls including cisplatin (50 mM) (24 h incubation), cells were collected by centrifugation for 5 min. Then cells were rinsed with cold water twice and resuspended in Annexin V-FITC binding buffer at a concentration of 1 × 10^6^ cells/mL. 5 mL of Annexin V-FITC was used to stain the cells. After 15 min of incubation at 25 °C, the cell suspension was centrifugated for 5 min and cells were resuspended in Annexin V-FITC binding buffer. Propidium iodide (5 mL) was added and the tubes were situated on ice and away from light. The fluorescence was measured using a flow cytometer. The results were analyzed by using FCSExpress software and represented as percentage of normal and apoptotic cells at various stages [49]. The percentage of apoptotic cells was calculated from the number of cells in sub-G1 phase, representing fragmented cell vesicles. The four areas in the diagrams stand for necrotic cells (Q1, positive for PI and negative for annexin/FITC, left square on the top), live cells (Q3, negative for annexin and PI, left square at the bottom), late apoptotic or necrotic cells (Q2, positive for annexin and PI, right square on the top) and apoptotic cells (Q4, negative for PI and positive for annexin, right square at the bottom), respectively. The experiment was repeated three times.

#### 3.2.4. Inhibition of AChE/BuChE Enzymes

In order to determine the acetyl- and butyrylcholinesterase enzyme inhibitions, experiments were carried out by making some changes in the method developed by Ellman [50]. Samples were prepared by dissolving in the appropriate solvent. Adding 20 μL enzyme solution (AChE or BuChE, 1 U/mL) and 10 μL sample solution to 2.4 mL buffer solution and it was incubated for 15 min at 37 °C. After incubation, 50 μL DTNB and 75 mM ATCI solution or 20 μL of 25 mM BTCI solution were added and allowed the mixture to incubate at room temperature for 30 min. For the control group, only 10 μL of solvent was added instead of the sample solution provided all protocols are the same. The absorbance values of the prepared mixture were measured spectrophotometrically using polystyrene forceps at 37 °C at 412 nm. Experiments were run in triplicate. The inhibition fractions of AChE yada BuChE enzymes were calculated using the following equation:

I(%) = 100 − (Abs_sample_ − Abs_control_) × 100

#### 3.2.5. Statistical Analysis

All data were recorded as mean ± standard error of mean (S.E.M). Results were analyzed by the one-way ANOVA plus Tukey’s test. *p*-values lower than 0.05 were considered significant (*p* < 0.05 *, *p* < 0.01 **, *p* < 0.001 ***).

## 4. Conclusions

In this study, we have synthesized novel eleven compounds combining two ring systems thiazoline and tetralin. *N*′-[3-cyclohexyl/phenyl-4-(substituted phenyl)thiazole-2(3*H*)-ylidene]-2-[(5,6,7,8-tetrahydronaphthalen-2-yl)oxy]acetohydrazide (**4a**–**4k**) derivatives have been tested to determine their anticancer potency. Compounds **4b** (IC_50_ = 69.2 μM) and **4d** IC_50_ = 71.8 μM showed the highest cytotoxicity against MCF-7 cells whereas compounds **4f**, **4g** and **4h** including 4-bromo, 4-chloro and 4-fluoro substitutions act at lower concentration than cisplatin. Compound **4d** inhibited DNA synthesis had the best potential of 47.75% at 35.9 μM concentration and 72.15% at 71.8 μM on MCF-7 cells. Compound **4h** inhibited DNA synthesis with a percentage of 48.97% at 24.3 μM and 50.78% at 48.6 μM on A549 cell line. With regard to flow cytometric analysis, compounds **4b** and **4e** exhibited 29.8% and 32.5% apoptosis (ealy + late apoptotic cell percentage), respectively against MCF-7 cell line, whereas cisplatin caused 29.9% apoptosis of the cells. Compounds **4a**, **4b**, **4f**, **4g**, **4h** and **4j** showed significant apoptosis levels whose apoptosis induction ratios were higher than cisplatin against A549 cell line. With regard to anticholinesterase activity of the compounds, compound **4h** showed the highest acetylcholinesterase inhibition with 49.92% and compounds **4j** with 44.96%, **4d** with 43.22%, **4f** with 42.87% and **4i** with 41.85% followed it.

## Figures and Tables

**Table 1:** The structures of the compounds (**4a**–**4k**).

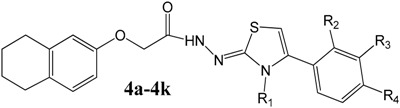				
Compounds	R_1_	R_2_	R_3_	R_4_
**4a**	Cyclohexyl	H	H	H
**4b**	Cyclohexyl	H	H	OCH_3_
**4c**	Phenyl	H	H	H
**4d**	Phenyl	H	H	CH_3_
**4e**	Phenyl	H	H	OCH_3_
**4f**	Phenyl	H	H	Br
**4g**	Phenyl	H	H	Cl
**4h**	Phenyl	H	H	F
**4i**	Phenyl	H	NO_2_	H
**4j**	Phenyl	H	H	NO_2_
**4k**	Phenyl	Cl	Cl	H

**Table 2:** IC_50_ values (µM) of the compounds and SI values against MCF-7, A549 and NIH/3T3 cell lines.

Compounds	MCF-7	A549	NIH/3T3	SI^MCF-7^	SI^A549^
**4a**	>1000	157.6 ± 14.8	206.1 ± 15.3	0.2	1.3
**4b**	69.2 ± 5.8	137.5 ± 7.2	55.7 ± 1.2	0.8	0.4
**4c**	296.7 ± 46.6	>1000	>1000	3.4	…
**4d**	71.8 ± 12.5	>1000	>1000	13.9	…
**4e**	91.4 ± 9.8	707.9 ± 83.3	>1000	10.9	1.4
**4f**	280.9 ± 56.2	54.3 ± 6.8	146.7 ± 5.4	0.5	2.7
**4g**	483.0 ± 31.2	28.6 ± 4.1	224.5 ± 20.5	0.5	7.8
**4h**	243.1 ± 14.9	48.6 ± 6.0	246.7 ± 12.2	1.0	5.1
**4i**	>1000	>1000	>1000	…	…
**4j**	>1000	49.0 ± 1.41	>1000	…	20.4
**4k**	152.7 ± 27.5	>1000	165.4 ± 11.0	1.1	…
**Cisplatin**	100.0 ± 3.3	108.3 ± 11.8	-	…	…

-: not tested; …:not calculated.

**Table 3:** Percents of typical quadrant analysis of Annexin V FITC/Propidium Iodide flow cytometry of MCF-7 and A549 cells treated with compounds and Cisplatin.

	Groups	% Early Apoptotic	% Late Apoptotic	% Viable	% Necrotic
**MCF-7 cell line**	**Control**	1.7	4.3	84.9	9.1
	**Compound 4b**	0.7	29.1	6.3	63.8
	**Compound 4d**	1.1	12.1	50.7	36.1
	**Compound 4e**	0.8	31.7	24.8	39.1
	**Compound 4k**	2.4	8.9	54.3	34.4
	**Cisplatin**	2.8	27.1	42.0	28.1
**A549 cell line**	**Control**	3.1	6.1	89.0	1.8
	**Compound 4a**	7.4	48.3	31.5	12.8
	**Compound 4b**	6.6	67.0	10.9	15.6
	**Compound 4f**	16.5	29.3	47.7	6.5
	**Compound 4g**	18.7	26.0	49.4	5.8
	**Compound 4h**	23.7	26.4	45.9	4.0
	**Compound 4j**	11.2	30.3	47.0	11.4
	**Cisplatin**	14.9	25.3	53.1	6.7

**Table 4:** % AChE and BuChE inhibitions of the compounds at 80 µg/mL concentration.

Compounds	AChE % Inhibition (80 µg/mL)	BuChE % Inhibition (80 µg/mL)
**4a**	38.26 ± 2.18	28 ± 1.88
**4b**	11.08 ± 1.48	15.89 ± 1.72
**4c**	19.30 ± 1.87	16.48 ± 0.08
**4d**	43.22 ± 1.56	33.19 ± 2.38
**4e**	10.81 ± 0.94	---
**4f**	42.87 ± 2.84	---
**4g**	24.37 ± 1.20	---
**4h**	49.92 ± 1.44	---
**4i**	41.85 ± 0.23	---
**4j**	44.96 ± 1.60	---
**4k**	15.19 ± 1.99	---
**Donepezil IC_50_ (µM)**	(11.8 ± 0.7) × 10^−3^	4.85 ± 0.55

---: no inhibition.

## Supplementary Materials

Supplementary materials are available online.

## References

[1] Zaharia V., Ignat A., Palibroda N., Ngameni B., Kuete V., Fokunang C.N., Moungang M.L., Ngadjui B.T. (2010). Synthesis of some p-toluenesulfonyl-hydrazinothiazoles and hydrazino-bis-thiazoles and their anticancer activity. Eur. J. Med. Chem..

[2] Ding H., Chen Z., Zhang C., Xin T., Wang Y., Song H., Jiang Y., Chen Y., Xu Y., Tan C. (2012). Synthesis and cytotoxic activity of some novel *N*-pyridinyl-2-(6-phenylimidazo[2,1-*b*]thiazol-3-yl)acetamide derivatives. Molecules.

[3] Liu Y., Zhang L., Gong J., Fang H., Liu A., Du G., Xu W. (2011). Design, synthesis, and biological activity of thiazole derivatives as novel influenza neuraminidase inhibitors. J. Enzym. Inhib. Med. Chem..

[4] Cai W.X., Liu A.L., Li Z.M., Dong W.L., Liu X.H., Sun N.B. (2016). Synthesis and anticancer activity of novel thiazole-5-carboxamide derivatives. Appl. Sci..

[5] Kashyap S.J., Kumar Garg V., Kumar Sharma P., Kumar N., Dudhe R., Kumar Gupta J. (2012). Thiazoles: Having diverse biological activities. Med. Chem. Res..

[6] Nastasa C., Tiperciuc B., Duma M., Benedec D., Oniga O. (2015). New hydrazones bearing thiazole scaffold: Synthesis, characterization, antimicrobial, and antioxidant investigation. Molecules.

[7] Bhole R.P., Bhusari K.P. (2011). Synthesis and antitumor activity of (4-hydroxyphenyl)[5-substituted alkyl/aryl)-2-thioxo-1,3,4-thiadiazol-3-yl]methanone and [(3,4-disubstituted)-1,3-thiazol-2ylidene]-4-hydroxybenzohydrazide. Med. Chem. Res..

[8] Sondhi S.M., Rani R., Gupta P.P., Agrawal S.K., Saxena A.K. (2009). Synthesis, anticancer, and anti-inflammatory activity evaluation of methanesulfonamide and amidine derivatives of 3,4-diaryl-2-imino-4-thiazolines. Mol. Divers..

[9] Makhaeva G.F., Boltneva N.P., Lushchekina S.V., Serebryakova O.G., Stupina T.S., Terentiev A.A., Serkov I.V., Proshin A.N., Bachurin S.O., Richardson R.J. (2016). Synthesis, molecular docking and biological evaluation of *N*,*N*-disubstituted 2-aminothiazolines as a new class of butyrylcholinesterase and carboxylesterase inhibitors. Bioorg. Med. Chem..

[10] Han F.S., Osajima H., Cheung M., Tokuyama H., Fukuyama T. (2007). Novel structural motifs consisting of chiral thiazolines: Synthesis, molecular recognition, and anticancer activity. Chemistry.

[11] Carmeli S., Moore R.E., Patterson G.L. (1991). Mirabazoles, minor tantazole-related cytotoxins from the terrestrial blue-green alga scytonema mirabil. Tetrahedron Lett..

[12] Igarashi Y., Asano D., Sawamura M., In Y., Ishida T., Imoto M. (2016). Ulbactins F and G, polycyclic thiazoline derivatives with tumor cell migration inhibitory activity from *Brevibacillus* sp.. Org. Lett..

[13] Marson C.M., Matthews C.J., Yiannaki E., Atkinson S.J., Soden P.E., Shukla L., Lamadema N., Thomas N.S. (2013). Discovery of potent, isoform-selective inhibitors of histone deacetylase containing chiral heterocyclic capping groups and a *N*-(2-aminophenyl)benzamide binding unit. J. Med. Chem..

[14] Patil P.A., Amnerkar N.D., Pathare S.S., Bhusari K.P. (2015). Docking study of p-hydroxybenzohydrazide derivatives as tyrosine kinase inhibitors and anticancer agents. J. Comput. Methods Mol. Des..

[15] Wang W., Zhao B., Xu C., Wu W. (2012). Synthesis and antitumor activity of the thiazoline and thiazine multithioether. Int. J. Org. Chem..

[16] Sondhi S.M., Johar M., Singh N., Dastidar S.G. (2004). Synthesis of biscoupled hemin-thiazoline derivatives and their anticancer activity evaluation. Indian J. Chem..

[17] Mahani N.M., Sabermahani F., Jahani P.M., Jalali N. (2015). A density function theory based quantitative structure activity relationships study of thiazoline derivatives as anticancer agents. Iran. J. Anal. Chem..

[18] Taher A.T., Khalil N.A., Ahmed E.M. (2011). Synthesis of novel isatin-thiazoline and isatin-benzimidazole conjugates as anti-breast cancer agents. Arch. Pharm. Res..

[19] Miller D.D., Dalton J.T., Gududuru V., Hurh E. (2005). Thiazoline analogs as cell proliferation inhibitors. Chem. Abstr..

[20] Ng R.A., Sui Z. (2005). Preparation of thiazoline derivatives as selective androgen receptor modulators (SARMs). Chem. Abstr..

[21] Pine M.J., Mirand E.A., Ambrus J.L., Bock F.G. (1983). Antitumor studies of 2- amino-2-thiazoline and other tumor-modifying agents. J. Med..

[22] Randazzo A., Bifulco G., Giannini C., Bucci M., Debitus C., Cirino G., Gomez-Paloma L. (2001). Halipeptins A and B: Two novel potent anti-inflammatory cyclic depsipeptides from the Vanuatu marine sponge Haliclona species. J. Am. Chem. Soc..

[23] Pérez-Perarnau A., Preciado S., Palmeri C.M., Moncunill-Massaguer C., Iglesias-Serret D., González-Gironès D.M., Miguel M., Karasawa S., Sakamoto S., Cosialls A.M. (2014). A trifluorinated thiazoline scaffold leading to pro-apoptotic agents targeting prohibitins. Angew. Chem. Int. Ed..

[24] Shih M.H., Ke F.Y. (2004). Syntheses and evaluation of antioxidant activity of sydnonyl substituted thiazolidinone and thiazoline derivatives. Bioorg. Med. Chem..

[25] Monneret C. (2001). Recent developments in the field of antitumour anthracyclines. Eur. J. Med. Chem..

[26] Cragg G.M., Newman D.J. (2005). Plants as a source of anti-cancer agents. J. Ethnopharmacol..

[27] Kusari S., Lamshöft M., Spiteller M. (2009). Aspergillus fumigatus Fresenius, an endophytic fungus from *Juniperus communis* L. Horstmann as a novel source of the anticancer pro-drug deoxypodophyllotoxin. J. Appl. Microbiol..

[28] Chaitramallu M., Devaraju P. (2014). Synthesis of organic nanoparticle of aryl tetralin compounds and study of their biological activities for targeted delivery system. Indian J. Adv. Chem. Sci..

[29] Chen H., Zuo S., Wang X., Tang X., Zhao M., Lu Y., Chen L., Liu J., Liu Y., Liu D. (2011). Synthesis of 4b-triazole-podophyllotoxin derivatives by azideealkyne cycloaddition and biological evaluation as potential antitumor agents. Eur. J. Med. Chem..

[30] Hamdy N.A., Gamel-Eldeen A.M., Abdel-Aziz H.A., El-Hussieny A.A., Fakhr I.A. (2010). Modulation of carcinogen metabolizing enzymes by new fused heterocycles pendant to 5,6,7,8-tetrahydronaphthalene derivatives. Eur. J. Med. Chem..

[31] Dong Y., Shi Q., Nakagawa-Goto K., Wua P., Bastow K.F., Morris-Natschke S.L., Lee K. (2009). Antitumor agents 269. Non-aromatic ring-A neotanshinlactone analog, TNO, asa new class of potent antitumor agents. Bioorg. Med. Chem. Lett..

[32] Gamal-Eldeen A.M., Hamdy N.A., Abdel-Aziz H.A., El-Hussieny A.A., Fakhr I.A. (2014). Induction of intrinsic apoptosis pathway in colon cancer HCT-116 cells by novel 2-substituted-5,6,7,8-tetrahydronaphthalene derivatives. Eur. J. Med. Chem..

[33] Hamdy N.A., Anwar M.M., Abu-Zied K.M., Awad H.M. (2013). Synthesis, tumor inhibitory and antioxidant activity of new polyfuncctionnaly 2-substituted-5,6,7,8-tetrahydronaphthalene derivatives containing pyridine, thioxopyridine and pyrazolopyridine moieties. Acta Pol. Pharm..

[34] Gurkan-Alp A.S., Mumcuoglu M., Andac C.A., Dayanc E., Cetin-Atalay R., Buyukbingol E. (2012). Synthesis, anticancer activities and molecular modeling studies of novel indole retinoid derivatives. Eur. J. Med. Chem..

[35] Al-Abdullah E.S. (2011). Synthesis and anticancer activity of some novel tetralin-6-yl-pyrazoline, 2-thioxopyrimidine, 2-oxopyridine, 2-thioxo-pyridine and 2-iminopyridine derivatives. Molecules.

[36] Ebeid M.Y., El Zahar M.I., Kamel M.M., Omar M.T., Anwar M.M. (2004). Novel 5,6,7,8-tetrahydronaphth-2-yl heterocycles of possible biological activity. Egypt. Pharmaceut. J..

[37] Gouhar R.S., Raafat E. (2015). Synthesis of (3-(naphthalen-1-yl)oxiran-2-yl)(5,6,7,8 tetrahydronaphthalen-2-yl)methanone and reaction with some nucleophiles for anticancer evaluation. Res. Chem. Intermed..

[38] Amin K.M., El-Zahar M.I., Anwar M.M., Kamel M.M., Mohamed M.H. (2009). Synthesis and anticancer actıvıty of novel tetralin-6-ylpyridine and tetralin-6-ylpyrimidine derivatives. Acta Pol. Pharm..

[39] Ali M.M., Hassan S.A. (2007). Role of some newly synthesized tetrahydronaphthalenthiazol derivatives as anticancer compounds. Int. J. Cancer Res..

[40] Solimana E.A., El-Zahar M.I., El-Masry A.H., Kamel M., Gohar R.S. (2010). Synthesis and anticancer evaluation of novel tetrahydronaphthalen-6-ylthiazole heterocycles against human HePG2 and MCF7 cell lines. Der Pharm. Chem..

[41] Čolović M.B., Krstić D.Z., Lazarević-Pašti T.D., Bondžić A.M., Vasić V.M. (2013). Acetylcholinesterase inhibitors: Pharmacology and toxicology. Curr. Neuropharmacol..

[42] Zovko A., Sepcic K., Turk T., Faimali M., Garaventa F., Chelossi E., Paleari L., Falugi C., Aluigi M.G., Angelini C. (2009). New aspects of the relationship between acethylcholinesterase activity and cancer I: Poly-Aps experiments. WSEAS Trans. Biol. Biomed..

[43] Ciftci G.A., Temel H.E., Yildirim S.U., Kaplancikli Z.A., Altintop M.D., Genc L. (2013). Apoptotic effects of some carbazole derivatives on lung carcinoma and glioma cell lines. Med. Chem. Res..

[44] Turan-Zitouni G., Ozdemir A., Kaplancikli Z.A., Revial G., Demirci F. (2011). Synthesis and antimicrobial activity evaluation of new hydrazide derivatives. Turk. J. Pharm. Sci..

[45] Turan-Zitouni G., Kaplancıklı Z.A., Erol K., Kılıç F.S. (1999). Synthesis and analgesic activity of some triazoles and triazolothiazines. II Farmaco.

[46] Berridge M.V., Herst P.M., Tan A.S. (2005). Tetrazolium dyes as tools in cell biology: New insights into their cellular reduction. Biotechnol. Annu. Rev..

[47] Mossmann T. (1983). Rapid colorimetric assay for cellular growth and survival: Application to proliferation and cytotoxicity assays. J. Immunol. Methods.

[48] Maghni K., Nicolescu O.M., Martin J.G. (1999). Suitability of cell metabolic colorimetric assays for assessment of CD4+ T cell proliferation: Comparison to 5-bromo-2-deoxyuridine (BrdU) ELISA. J. Immunol. Methods.

[49] Lowe S.W., Lin A.W. (2000). Apoptosis in cancer. Carcinogenesis.

[50] Ellman G.L., Courtney K.D., Andres V., Feather-Stone R.M. (1961). A new and rapid colorimetric determination of acetylcholinesterase activity. Biochem. Pharmacol..

