# Supplementary material for: New Thiazoline-Tetralin Derivatives and Biological Activity Evaluation
Source: Molecules. 2018 Jan 10;23(1):135. doi: 10.3390/molecules23010135 (PMC6017121; doi:10.3390/molecules23010135)

Supplementary document

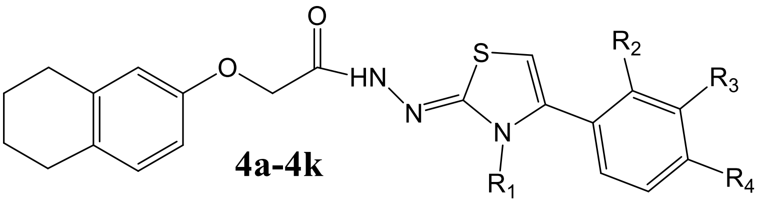

4a-4k

**Compounds**	**R_1**	**R_2**	**R_3**	**R_4**
**4a**	Cyclohexyl	H	H	H
**4b**	Cyclohexyl	H	H	OCH_3
**4c**	Phenyl	H	H	H
**4d**	Phenyl	H	H	CH_3
**4e**	Phenyl	H	H	OCH_3
**4f**	Phenyl	H	H	Br
**4g**	Phenyl	H	H	Cl
**4h**	Phenyl	H	H	F
**4i**	Phenyl	H	NO_2	H
**4j**	Phenyl	H	H	NO_2
**4k**	Phenyl	Cl	Cl	H

-IR spectrum of the compound 4a

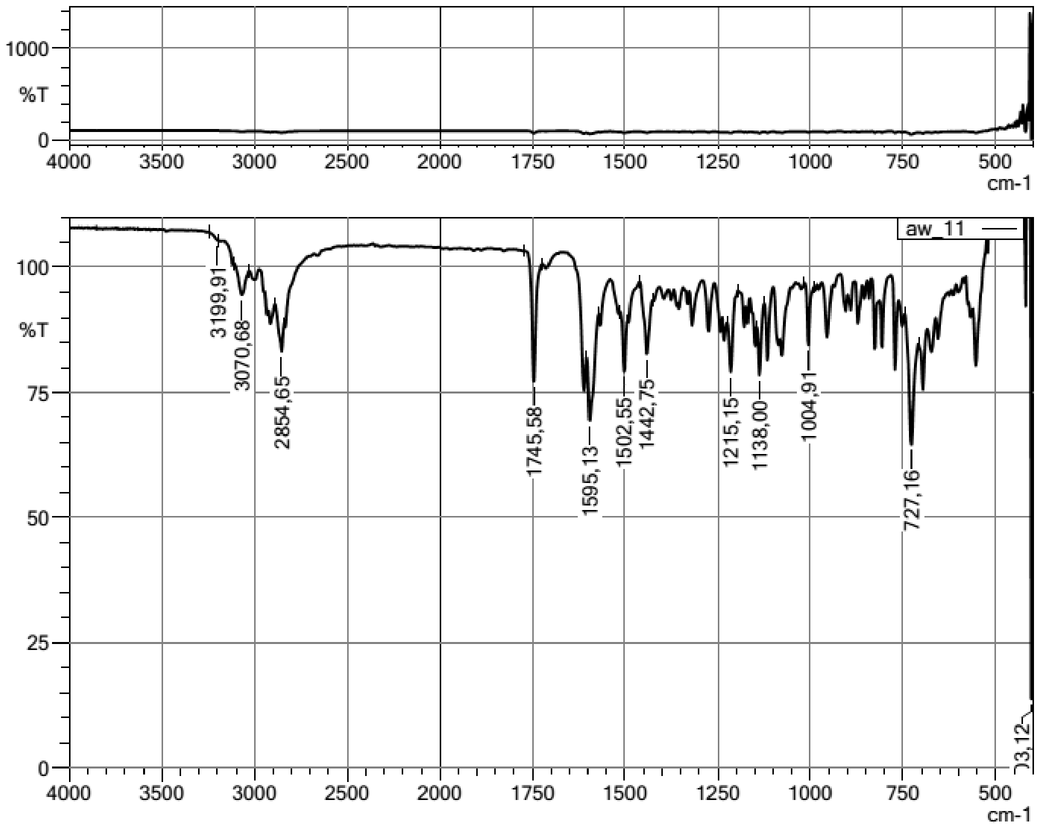

- HRMS spectrum of the compound4a

Data File: C:\LabSolutions\Data\Analiz\Lyuttas\AW-1_1.lcd

Elmt	Val.	Min	Max	Elmt	Val.	Min	Max	Elmt	Val.	Min	Max	Elmt	Val.	Min	Max	Use Adduct
H	1	20	35	O	2	2	4	Cl	1	0	1	I	3	0	0	H
C	4	25	30	F	1	0	1	Br	1	0	1					
N	3	3	4	S	2	1	1	Ru	2	0	0					

Error Margin (ppm): 5
HC Ratio: unlimited
Max Isotopes: 3
MSn Iso RI (%): 10.00

DBE Range: not fixed
Apply N Rule: yes
Isotope RI (%): 1.00
MSn Logic Mode: AND

Electron Ions: both
Use MSn Info: no
Isotope Res: 10000
Max Results: 500

Event#: 1 MS(E+) Ret. Time : 4.840 -> 4.960 Scan# : 727 -> 745

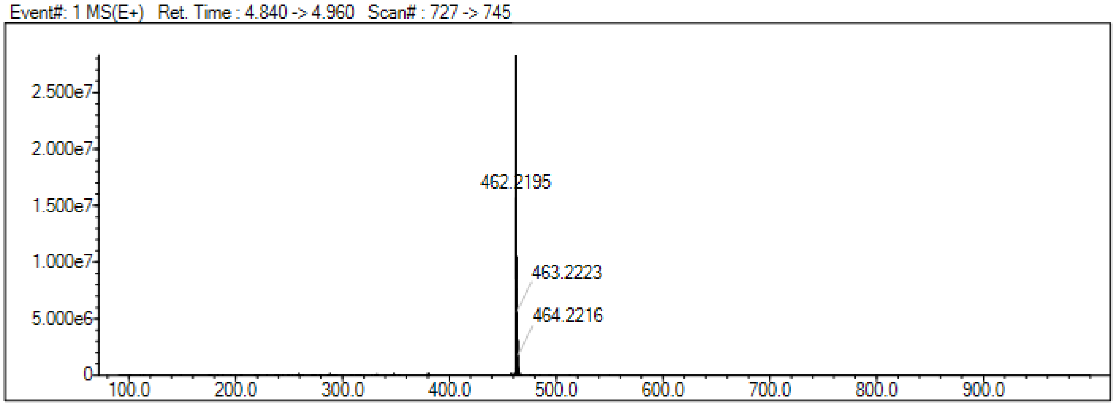

-^{1}H NMR spectrum of the compound 4a

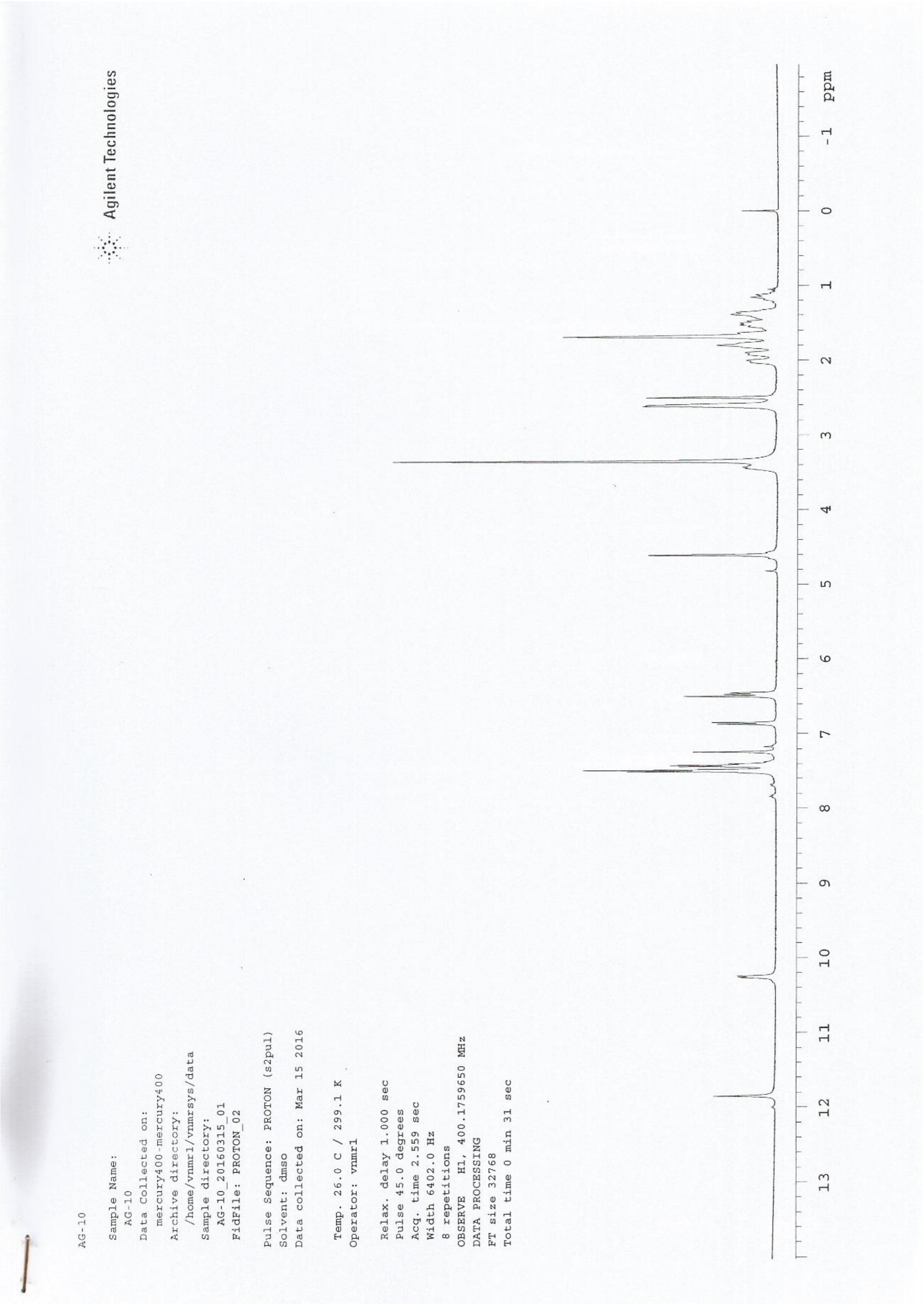

-^{13}C NMR spectrum of the compound 4a

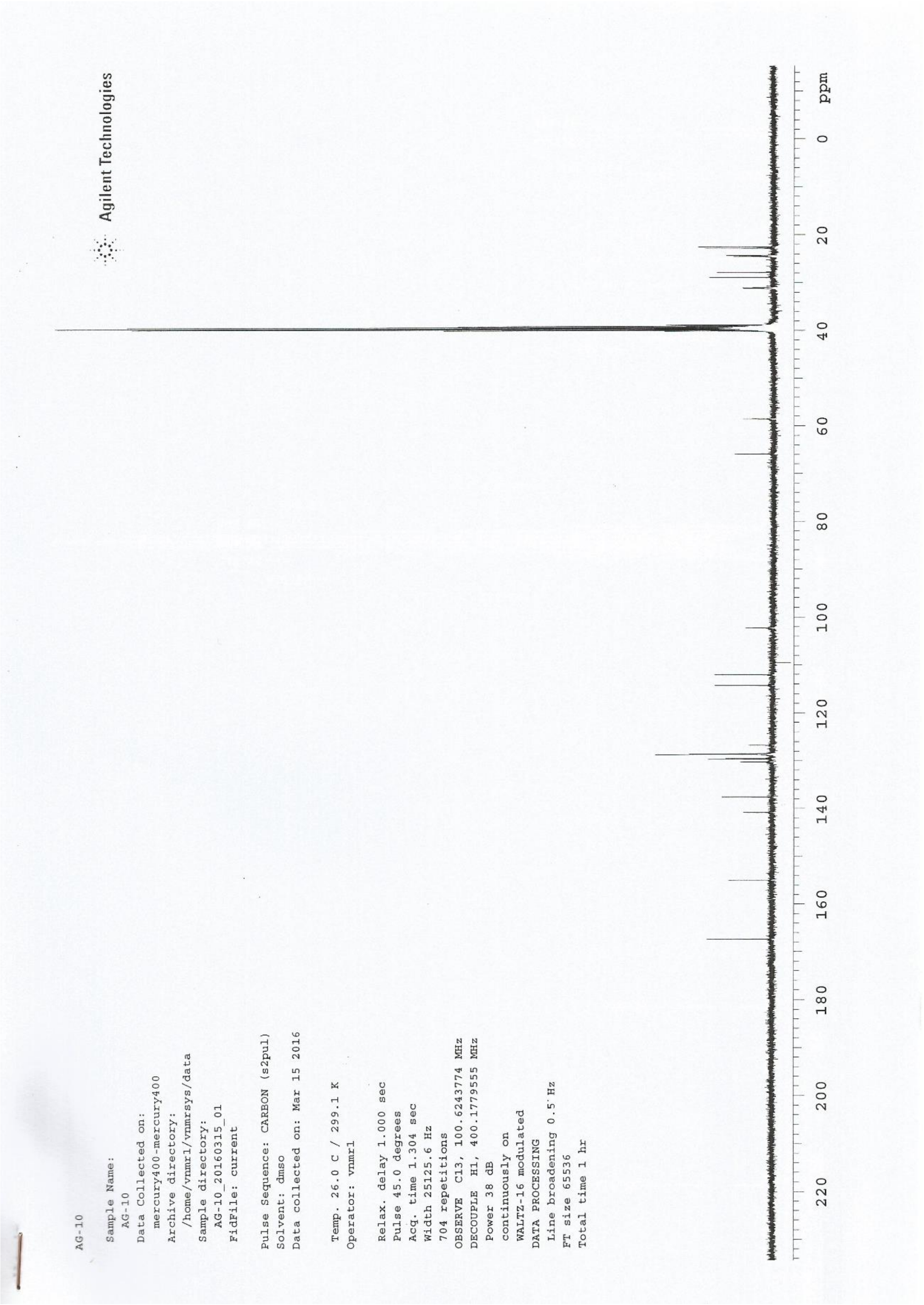

-IR spectrum of the compound 4b

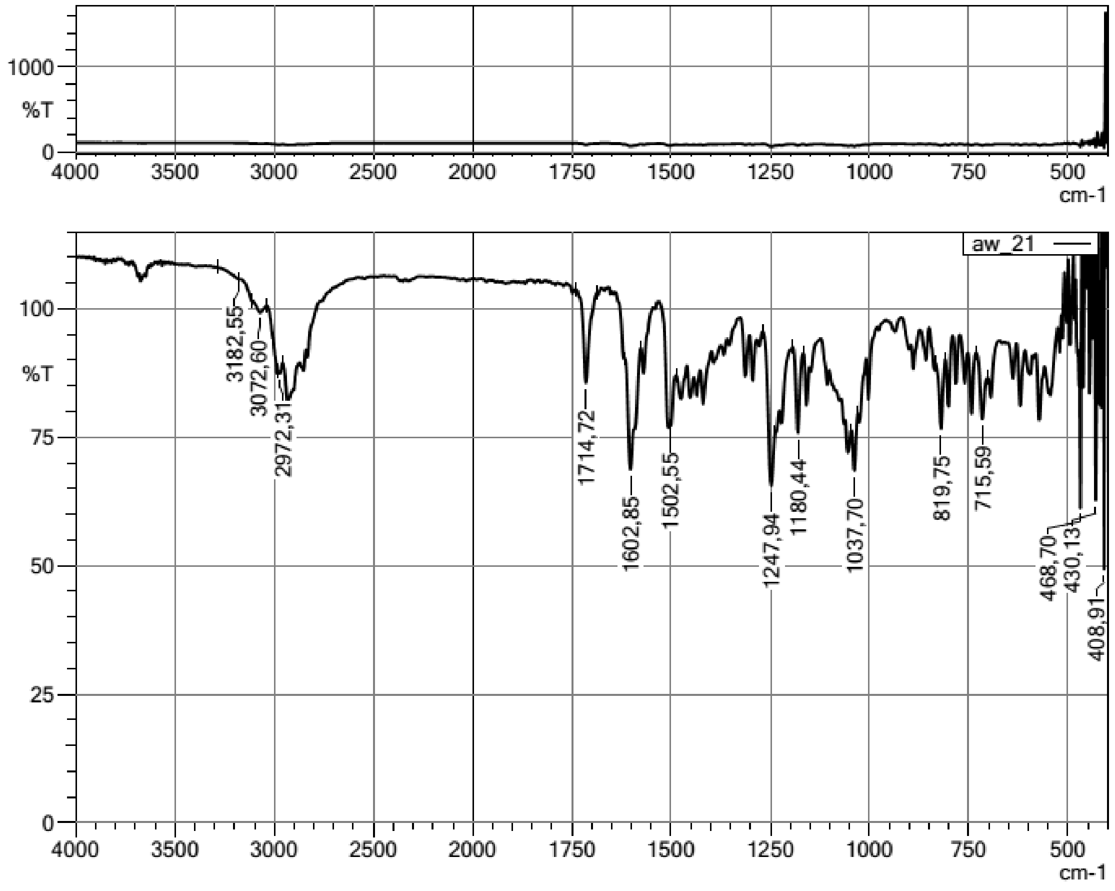

-HRMS spectrum of the compound 4b

Data File: C:\LabSolutions\Data\Analiz\Lyuttas\AW-2_2.lcd

Elmt	Val.	Min	Max	Elmt	Val.	Min	Max	Elmt	Val.	Min	Max	Elmt	Val.	Min	Max	Use Adduct
H	1	20	35	O	2	2	4	Cl	1	0	1	I	3	0	0	H
C	4	25	30	F	1	0	1	Br	1	0	1					
N	3	3	4	S	2	1	1	Ru	2	0	0					

Error Margin (ppm): 5
HC Ratio: unlimited
Max Isotopes: 3
MSn Iso RI (%): 10.00

DBE Range: not fixed
Apply N Rule: yes
Isotope RI (%): 1.00
MSn Logic Mode: AND

Electron Ions: both
Use MSn Info: no
Isotope Res: 10000
Max Results: 500

Event#: 1 MS(E+) Ret. Time : 4.933 -> 5.107 Scan# : 741 -> 767

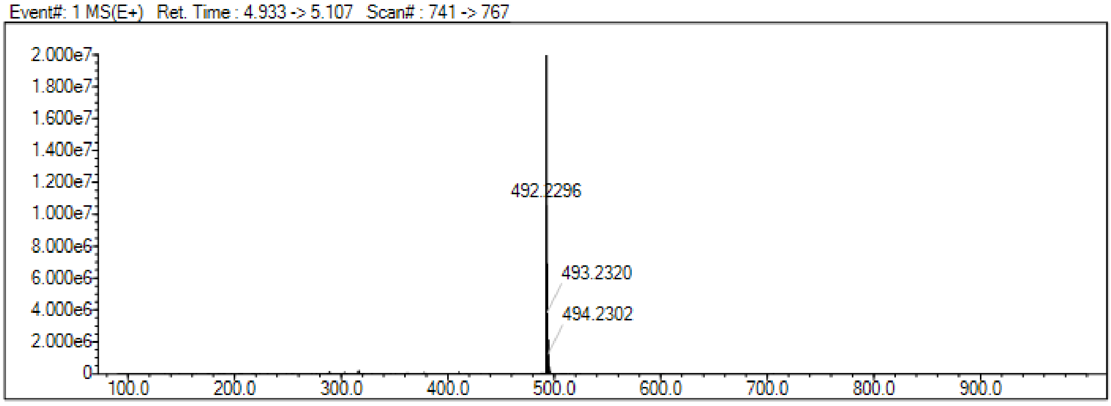

-^{1}H NMR spectrum of the compound 4b

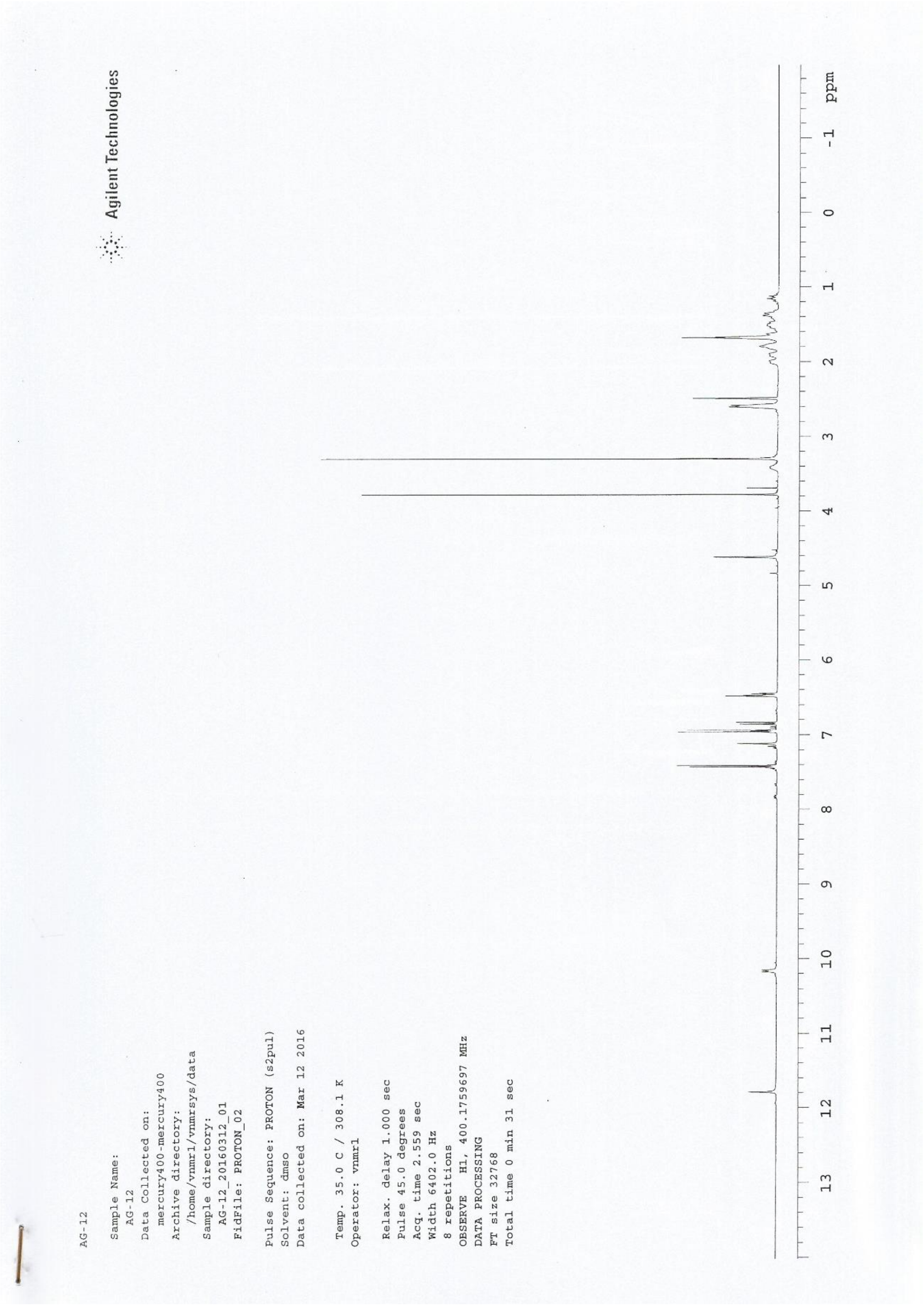

-^{13}C NMR spectrum of the compound 4b

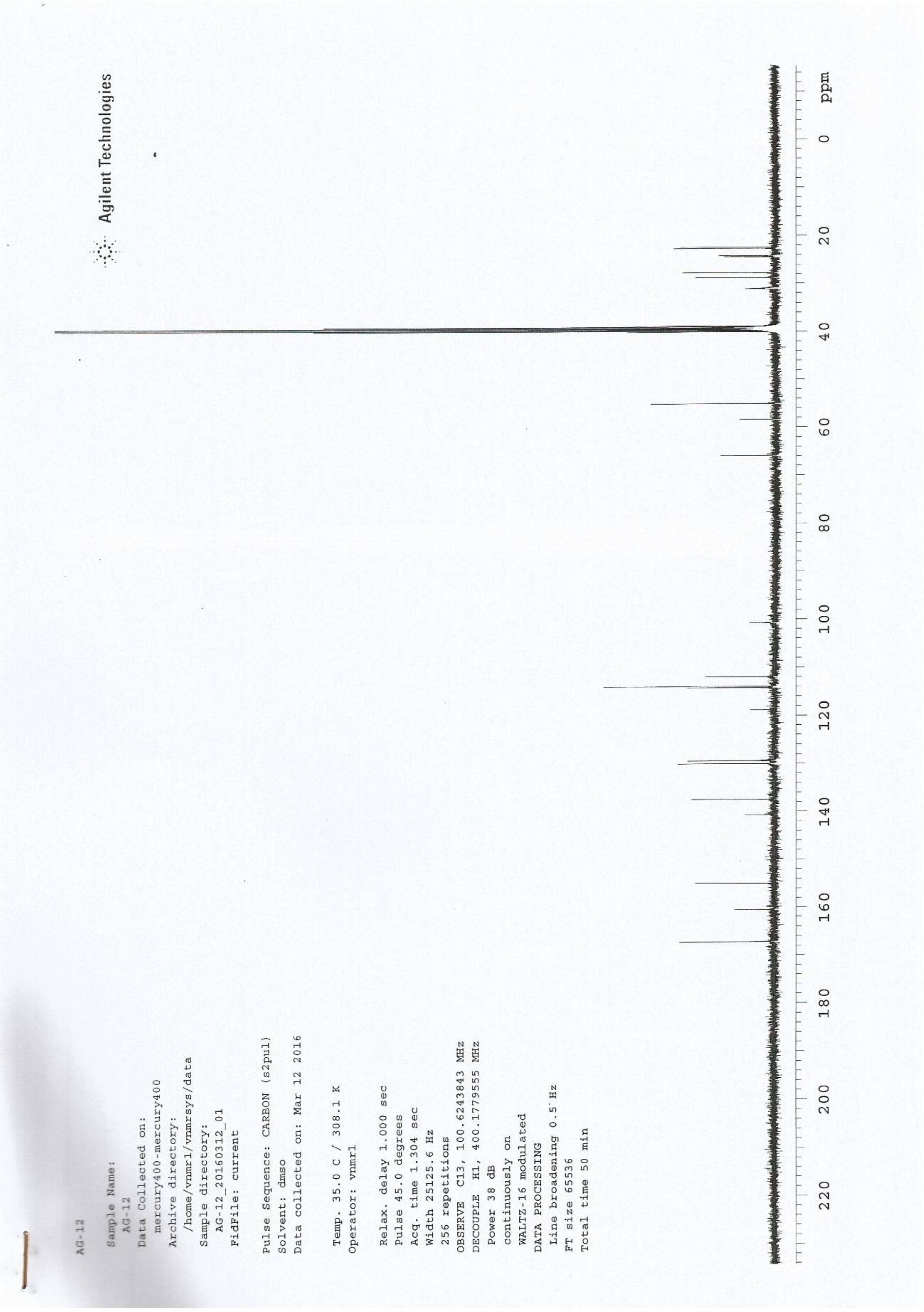

- IR spectrum of the compound 4c

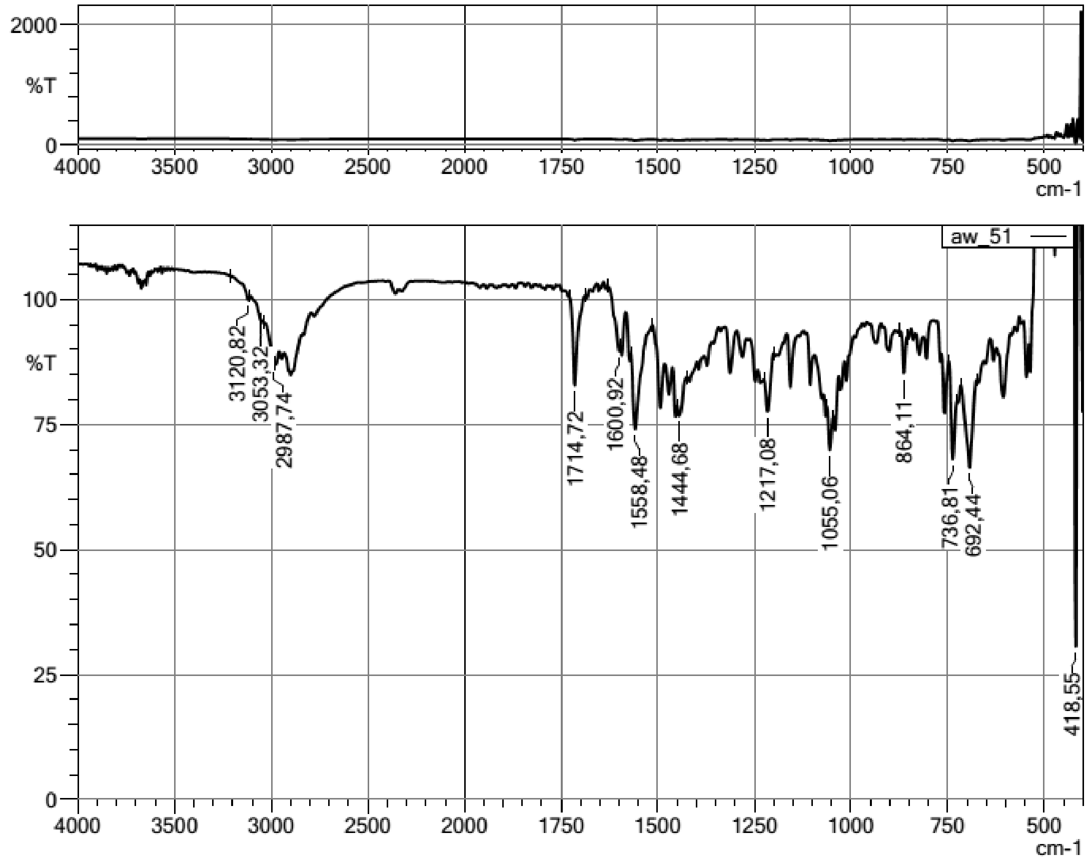

HRMS spectrum of the compound4c

Data File: C:\LabSolutions\Data\Analiz\Lyuttas\AW-5_3.lcd

Elmt	Val.	Min	Max	Elmt	Val.	Min	Max	Elmt	Val.	Min	Max	Elmt	Val.	Min	Max	Use Adduct
H	1	20	35	O	2	2	4	Cl	1	0	1	I	3	0	0	H
C	4	25	30	F	1	0	1	Br	1	0	1					
N	3	3	4	S	2	1	1	Ru	2	0	0					

Error Margin (ppm): 5
HC Ratio: unlimited
Max Isotopes: 3
MSn Iso RI (%): 10.00

DBE Range: not fixed
Apply N Rule: yes
Isotope RI (%): 1.00
MSn Logic Mode: AND

Electron Ions: both
Use MSn Info: no
Isotope Res: 10000
Max Results: 500

Event#: 1 MS(E+) Ret. Time : 7.320 -> 7.507 Scan# : 1099 -> 1127

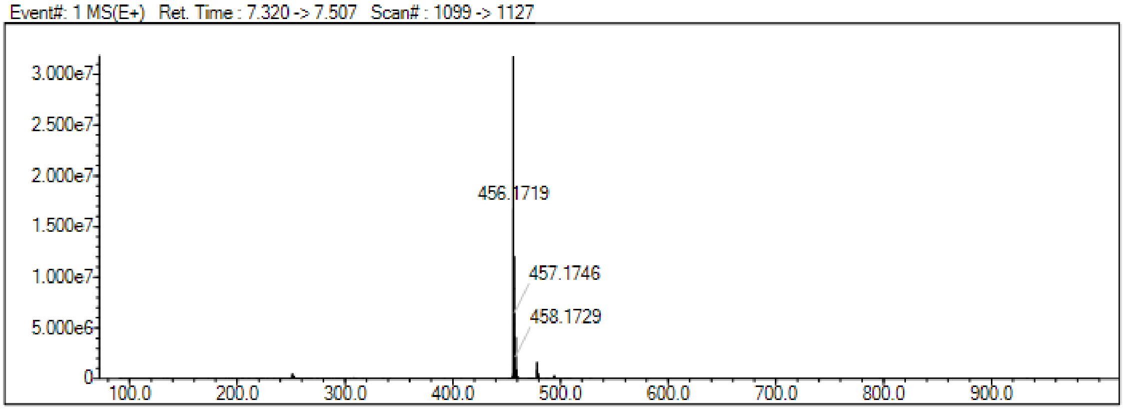

-^{1}H NMR spectrum of the compound 4c

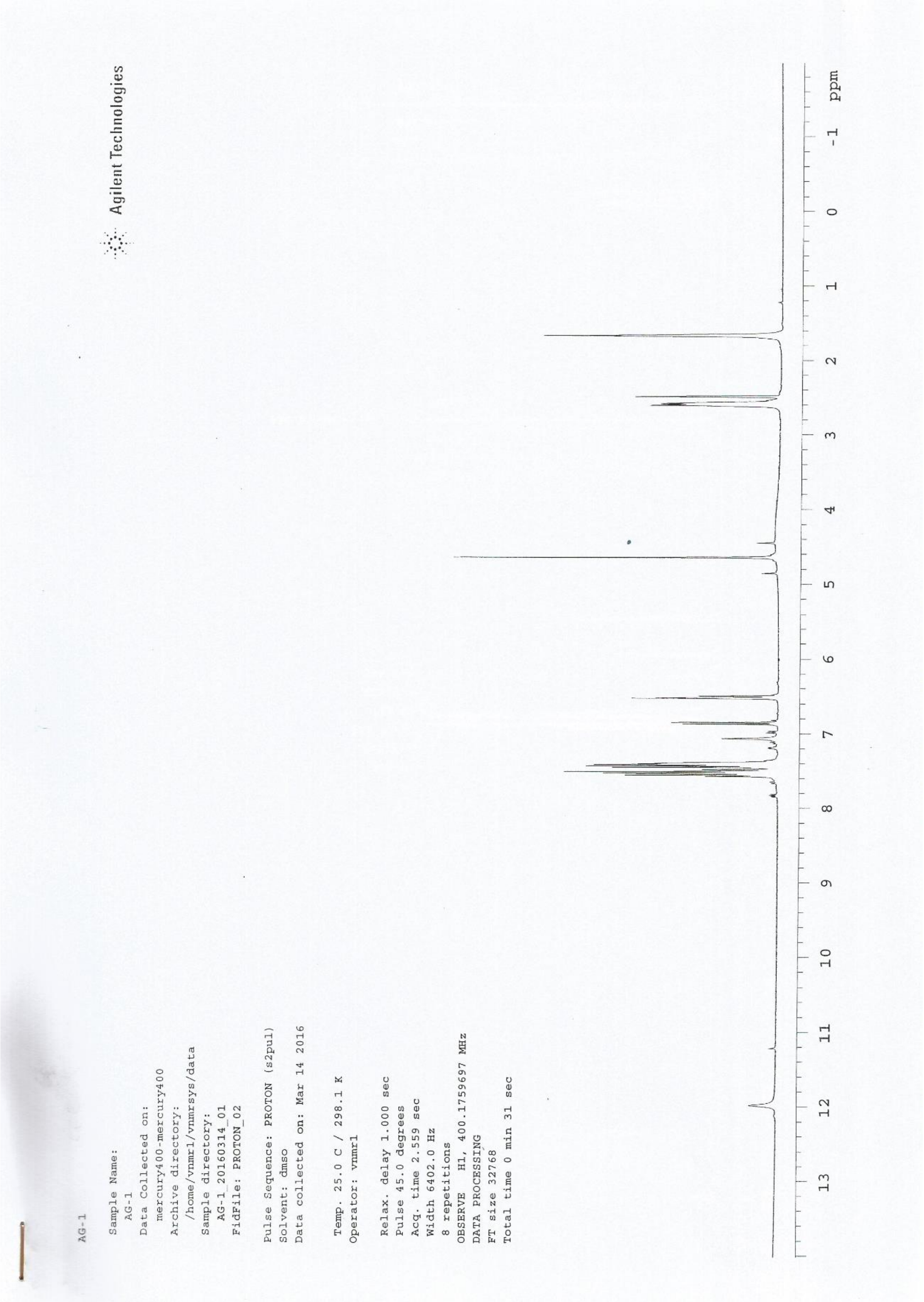

-^{13}C NMR spectrum of the compound 4c

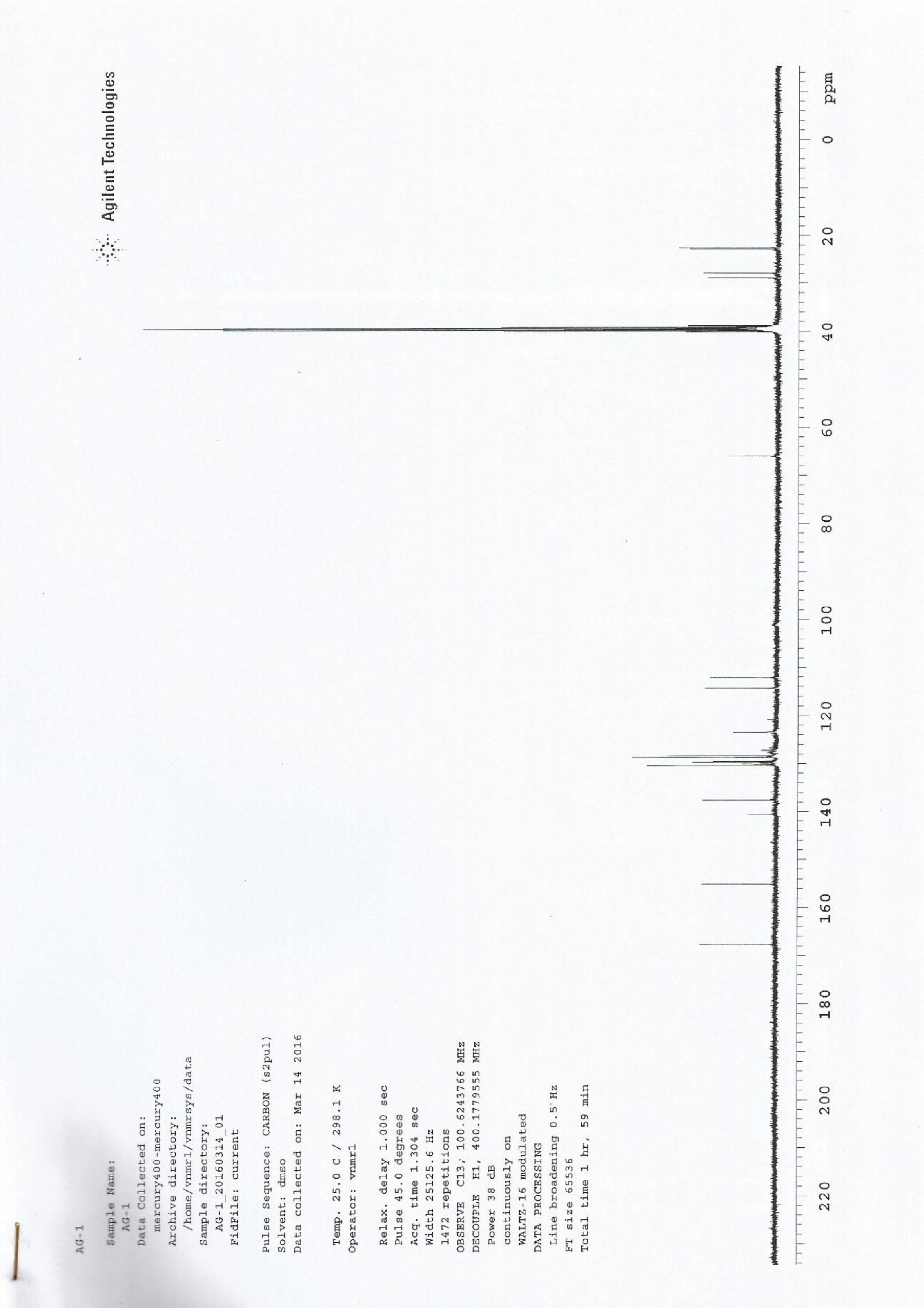

- IR spectrum of the compound 4d

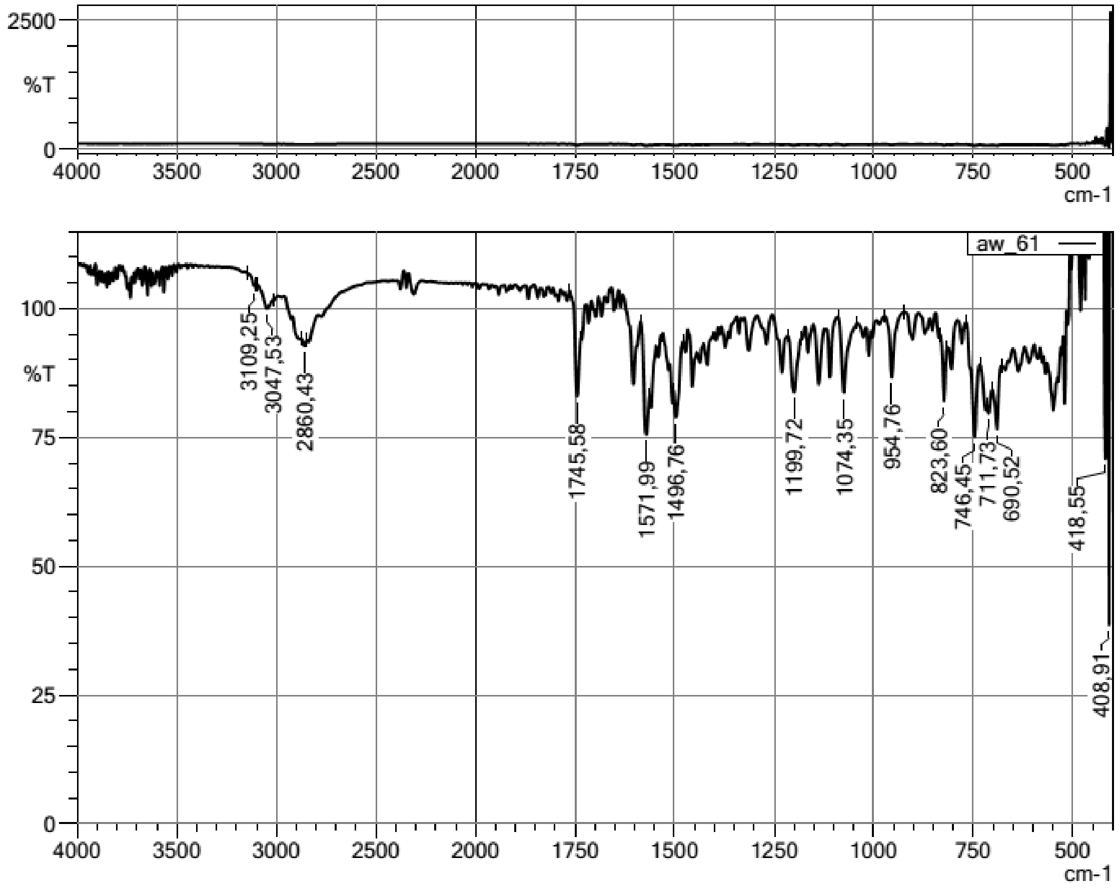

-HRMS spectrum of the compound 4d

Data File: C:\LabSolutions\Data\Analiz\Lyuttas\AW-6_4.lcd

Elmt	Val.	Min	Max	Elmt	Val.	Min	Max	Elmt	Val.	Min	Max	Elmt	Val.	Min	Max	Use Adduct
H	1	20	35	O	2	2	4	Cl	1	0	1	I	3	0	0	H
C	4	26	30	F	1	0	1	Br	1	0	1					
N	3	3	4	S	2	1	1	Ru	2	0	0					

Error Margin (ppm): 5
HC Ratio: unlimited
Max Isotopes: 3
MSn Iso RI (%): 10.00

DBE Range: not fixed
Apply N Rule: yes
Isotope RI (%): 1.00
MSn Logic Mode: AND

Electron Ions: both
Use MSn Info: no
Isotope Res: 10000
Max Results: 500

Event#: 1 MS(E+) Ret. Time : 7.520 -> 7.733 Scan# : 1129 -> 1161

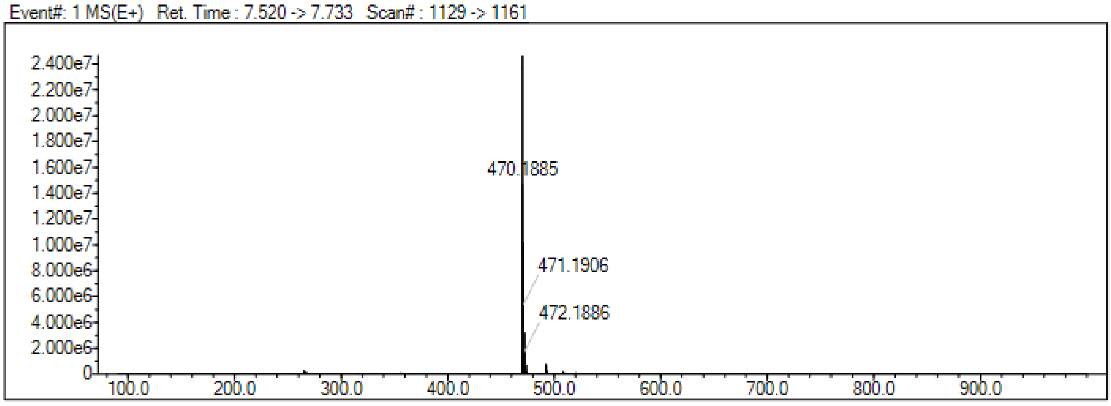

-^{1}H NMR spectrum of the compound 4d

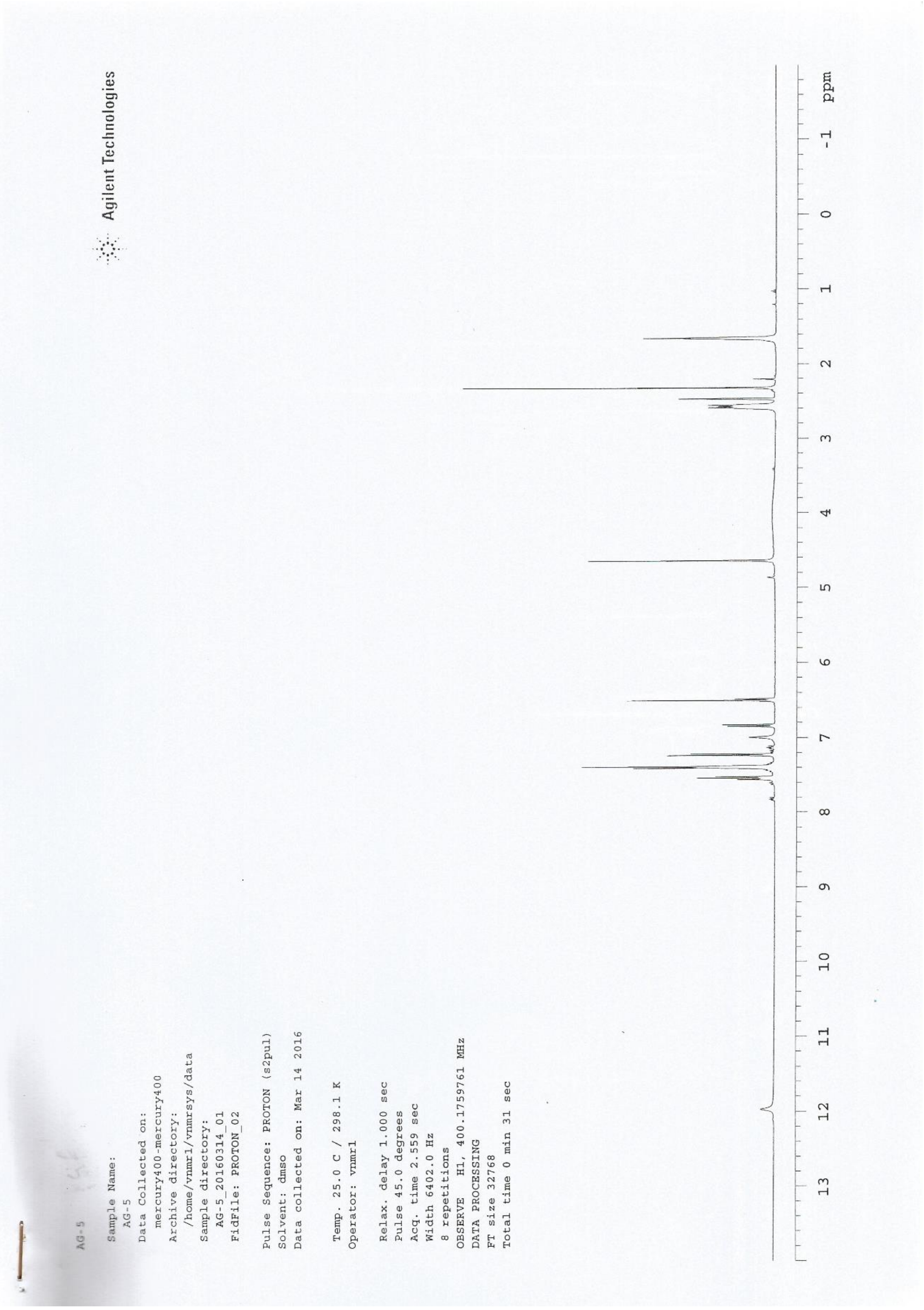

-^{13}C NMR spectrum of the compound 4d

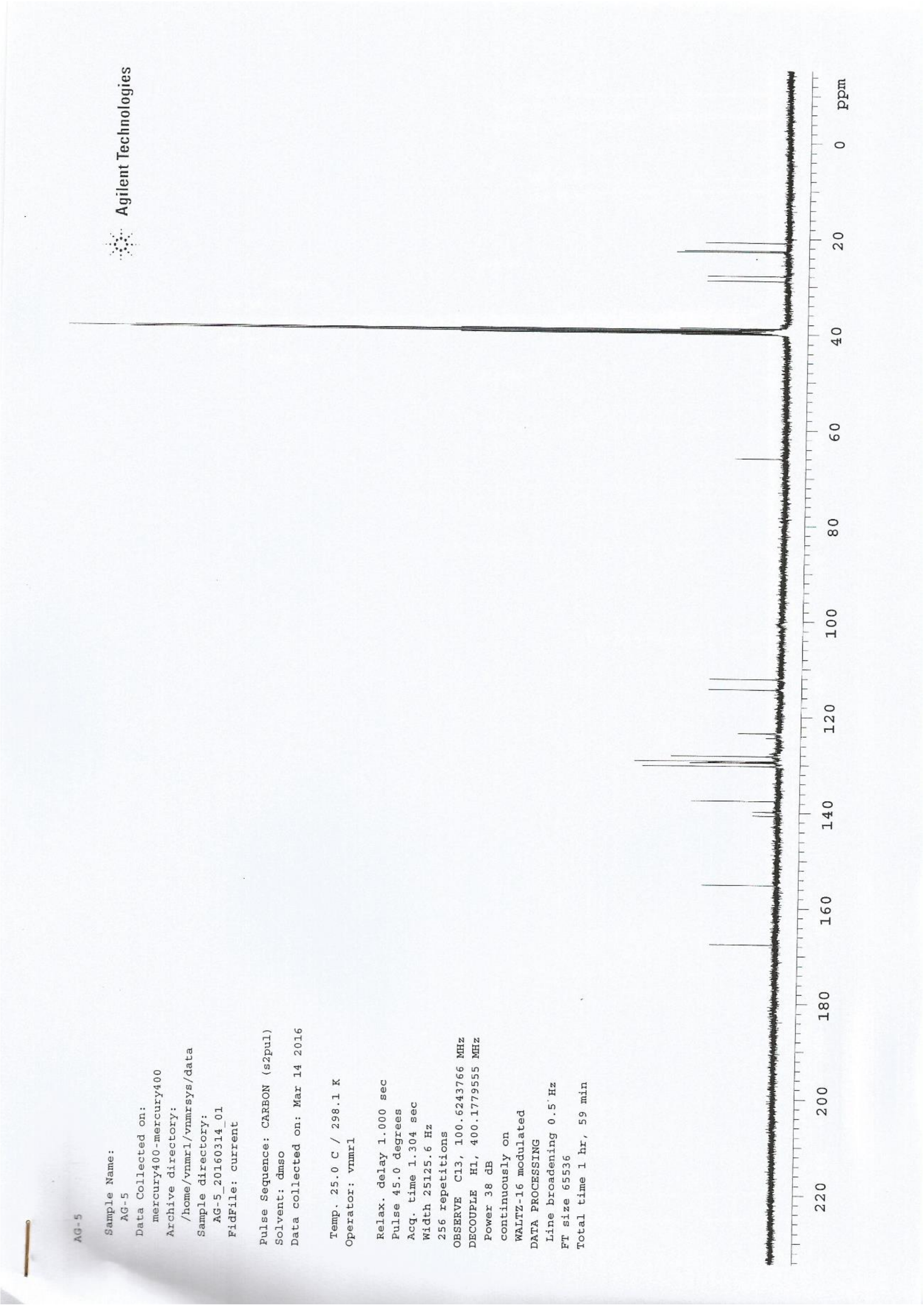

-IR spectrum of the compound 4e

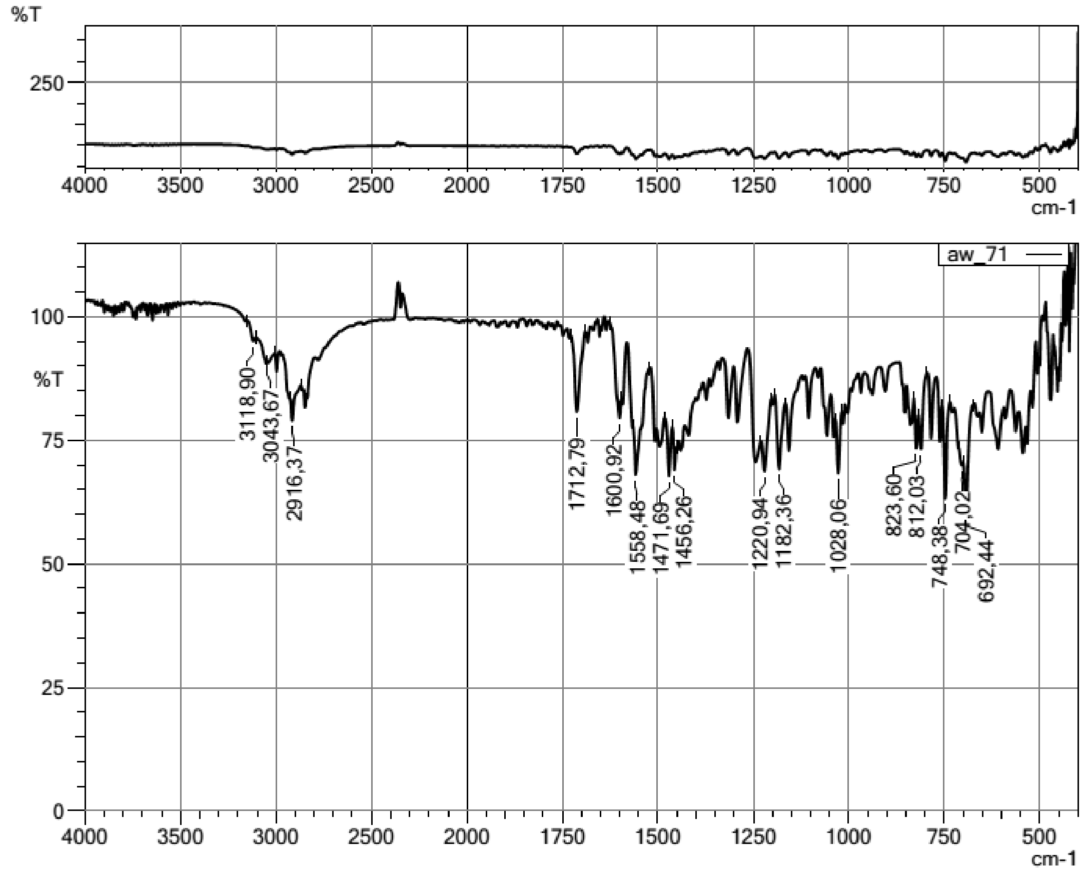

-HRMS spectrum of the compound 4e

Data File: C:\LabSolutions\Data\Analiz\Lyuttas\AW-7_5.lcd

Elmt	Val.	Min	Max	Elmt	Val.	Min	Max	Elmt	Val.	Min	Max	Elmt	Val.	Min	Max	Use Adduct
H	1	20	35	O	2	2	4	Cl	1	0	1	I	3	0	0	H
C	4	26	30	F	1	0	1	Br	1	0	1					
N	3	3	4	S	2	1	1	Ru	2	0	0					

Error Margin (ppm): 5
HC Ratio: unlimited
Max Isotopes: 3
MSn Iso RI (%): 10.00

DBE Range: not fixed
Apply N Rule: yes
Isotope RI (%): 1.00
MSn Logic Mode: AND

Electron Ions: both
Use MSn Info: no
Isotope Res: 10000
Max Results: 500

Event#: 1 MS(E+) Ret. Time : 7.067 -> 7.387 Scan# : 1061 -> 1109

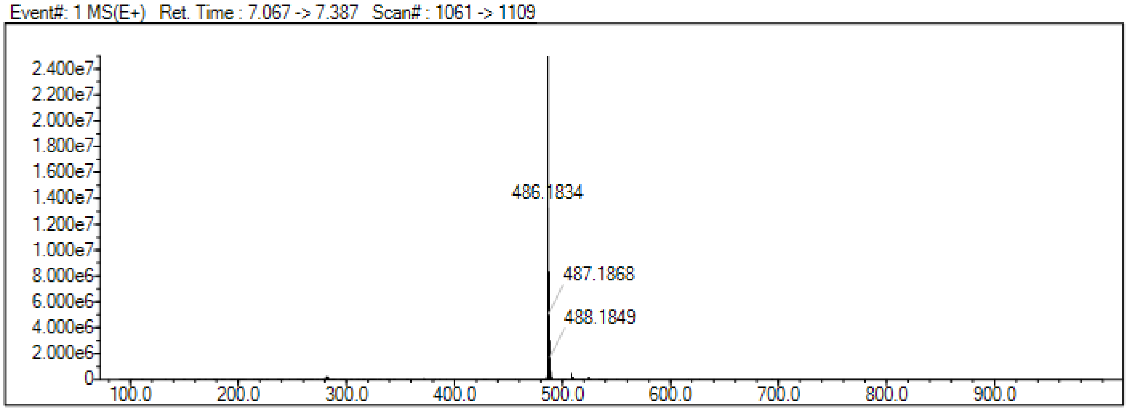

-^{1}H NMR spectrum of the compound 4e

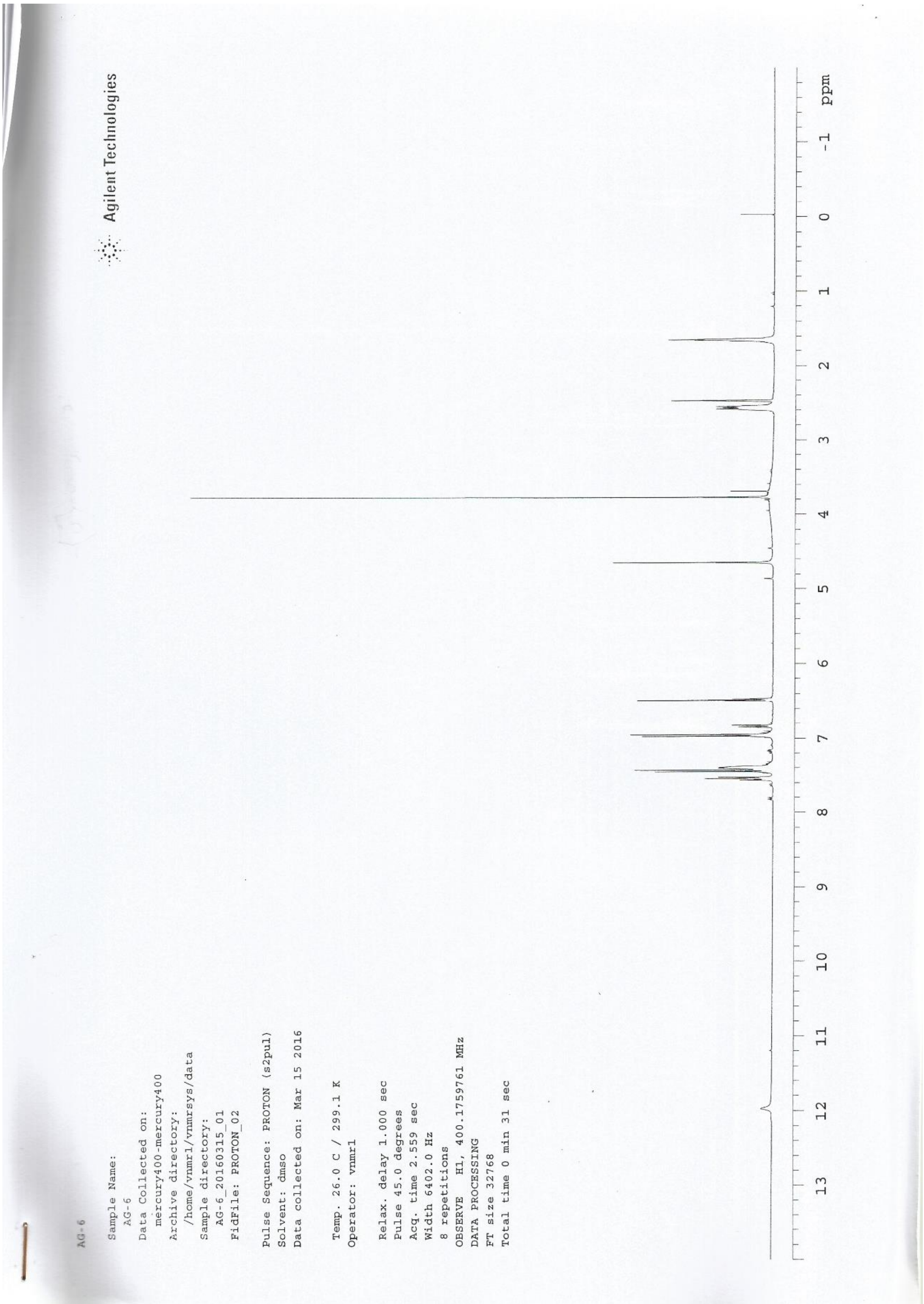

^{13}C NMR spectrum of the compound 4e

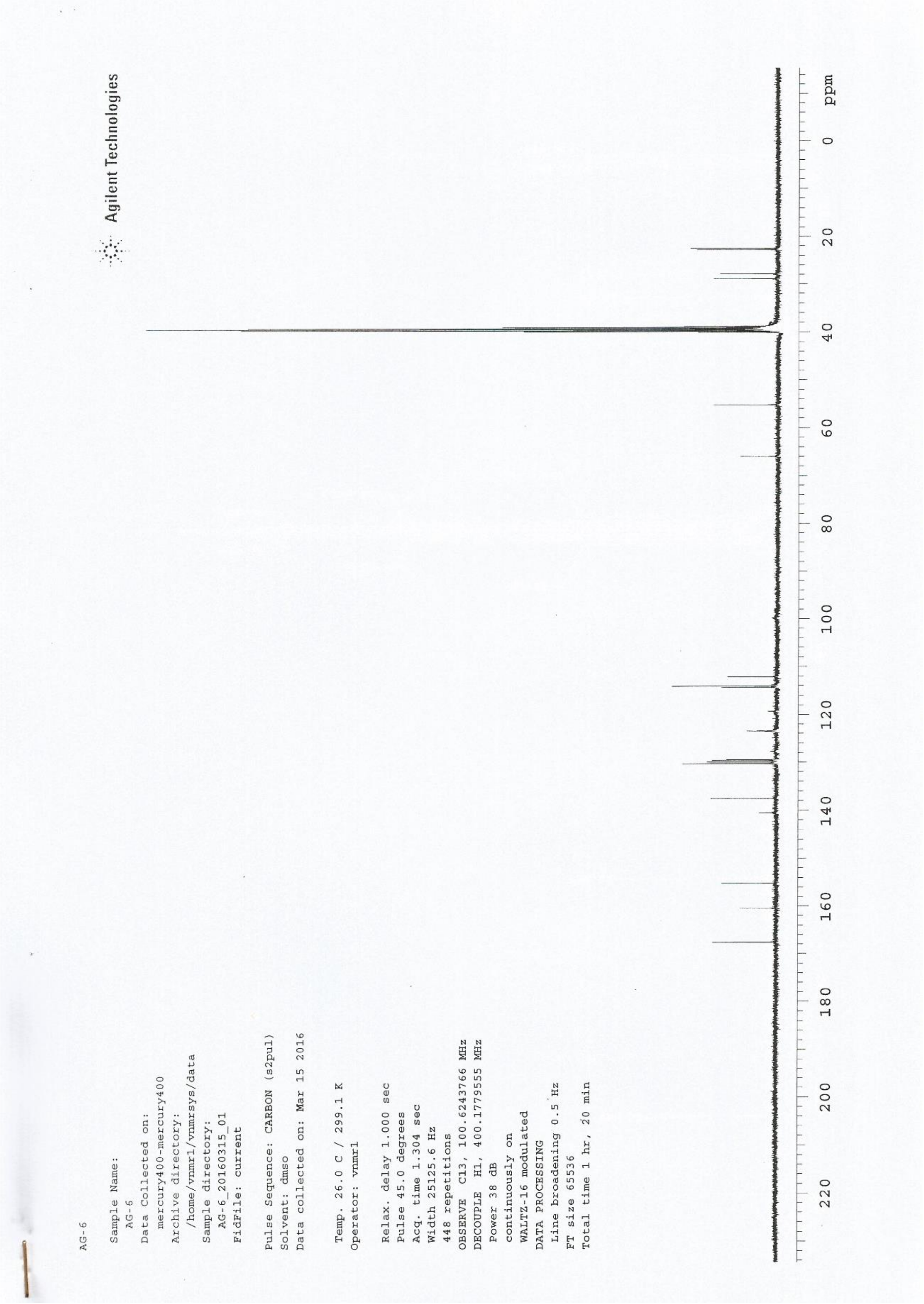

-IR spectrum of the compound 4f

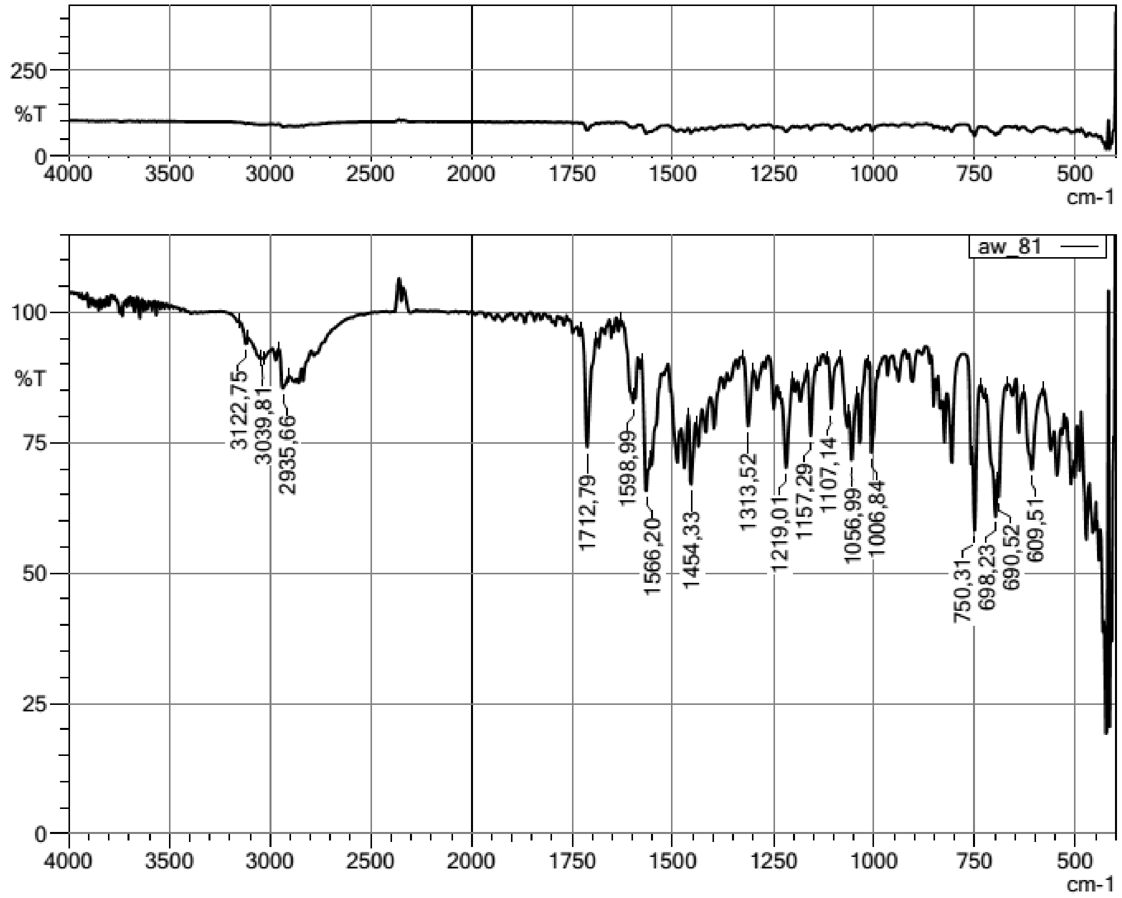

-HRMS spectrum of the compound 4f

Data File: C:\LabSolutions\Data\Analiz\Lyuttas\AW-8_9.lcd

Elmt	Val.	Min	Max	Elmt	Val.	Min	Max	Elmt	Val.	Min	Max	Elmt	Val.	Min	Max	Use Adduct
H	1	20	35	O	2	2	4	Cl	1	0	1	I	3	0	0	H
C	4	26	30	F	1	0	1	Br	1	0	1					
N	3	3	4	S	2	1	1	Ru	2	0	0					

Error Margin (ppm): 5
HC Ratio: unlimited
Max Isotopes: 3
MSn Iso RI (%): 10.00

DBE Range: not fixed
Apply N Rule: yes
Isotope RI (%): 1.00
MSn Logic Mode: AND

Electron Ions: both
Use MSn Info: no
Isotope Res: 10000
Max Results: 500

Event#: 1 MS(E+) Ret. Time : 7.987 -> 8.187 Scan# : 1199 -> 1229

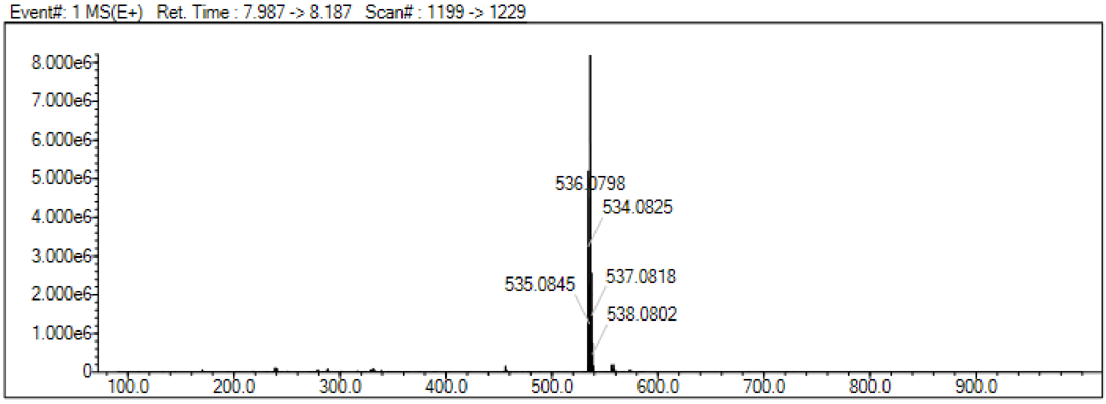

-^{1}H NMR spectrum of the compound 4f

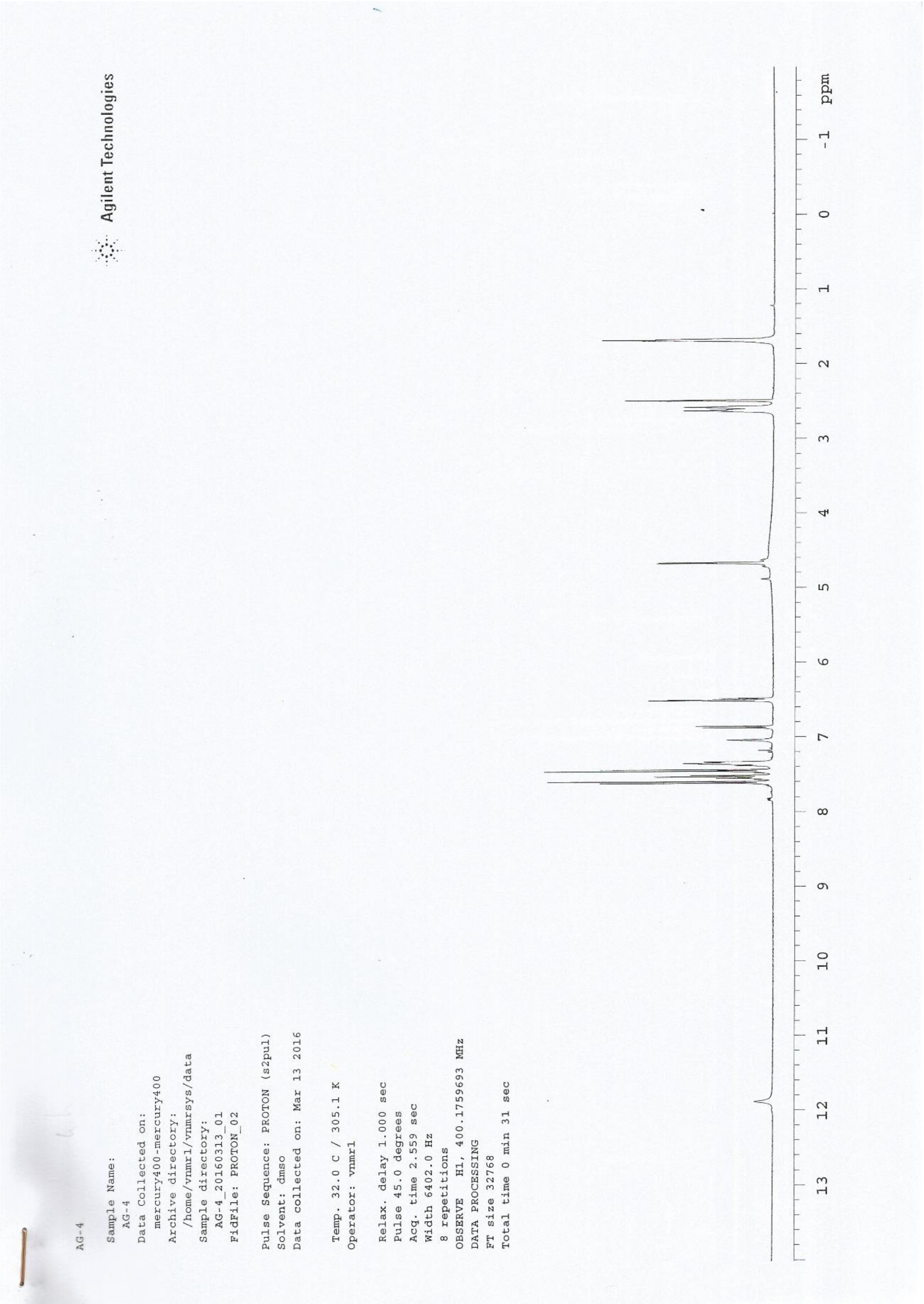

-^{13}C NMR spectrum of the compound 4f

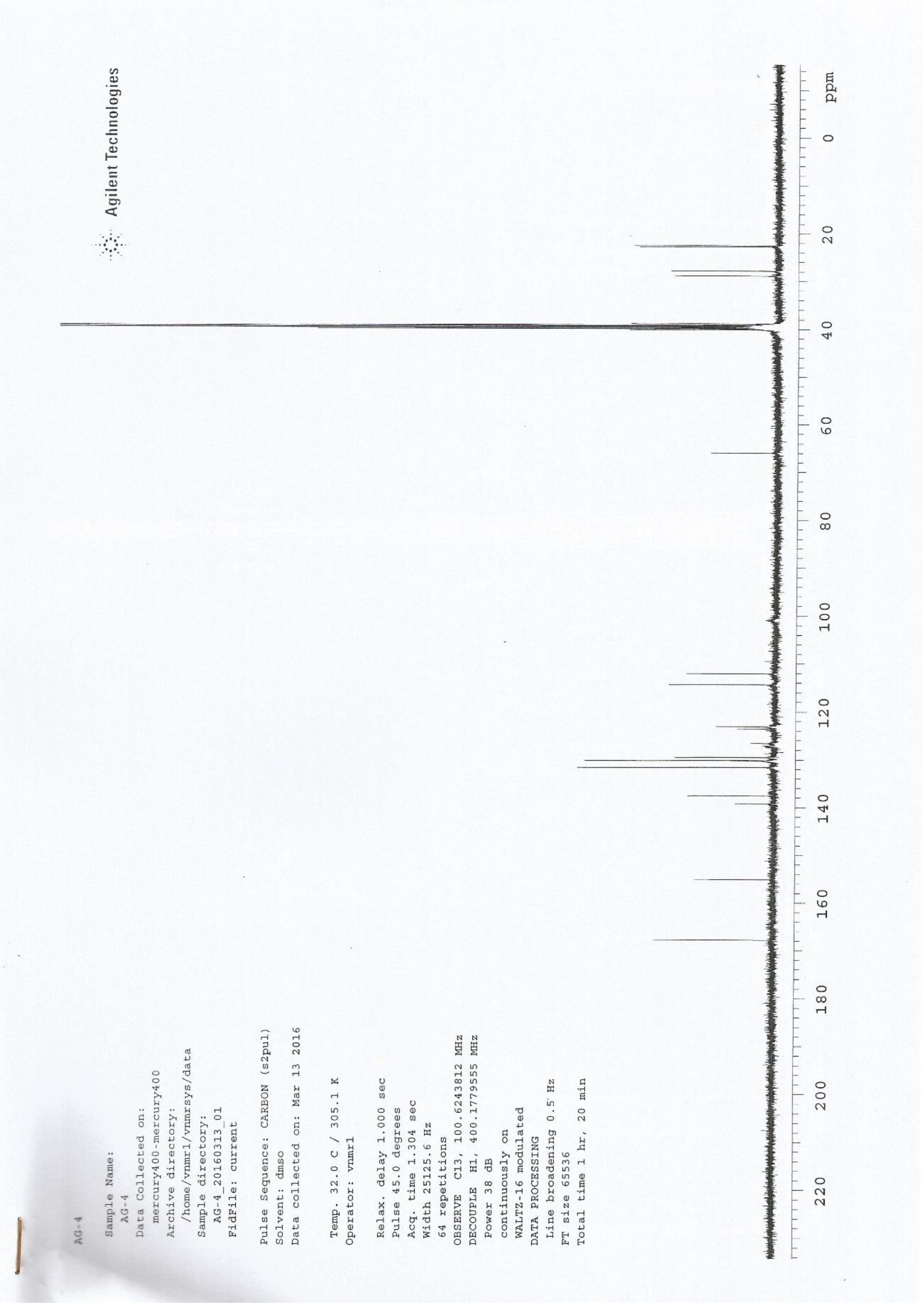

-IR spectrum of the compound 4g

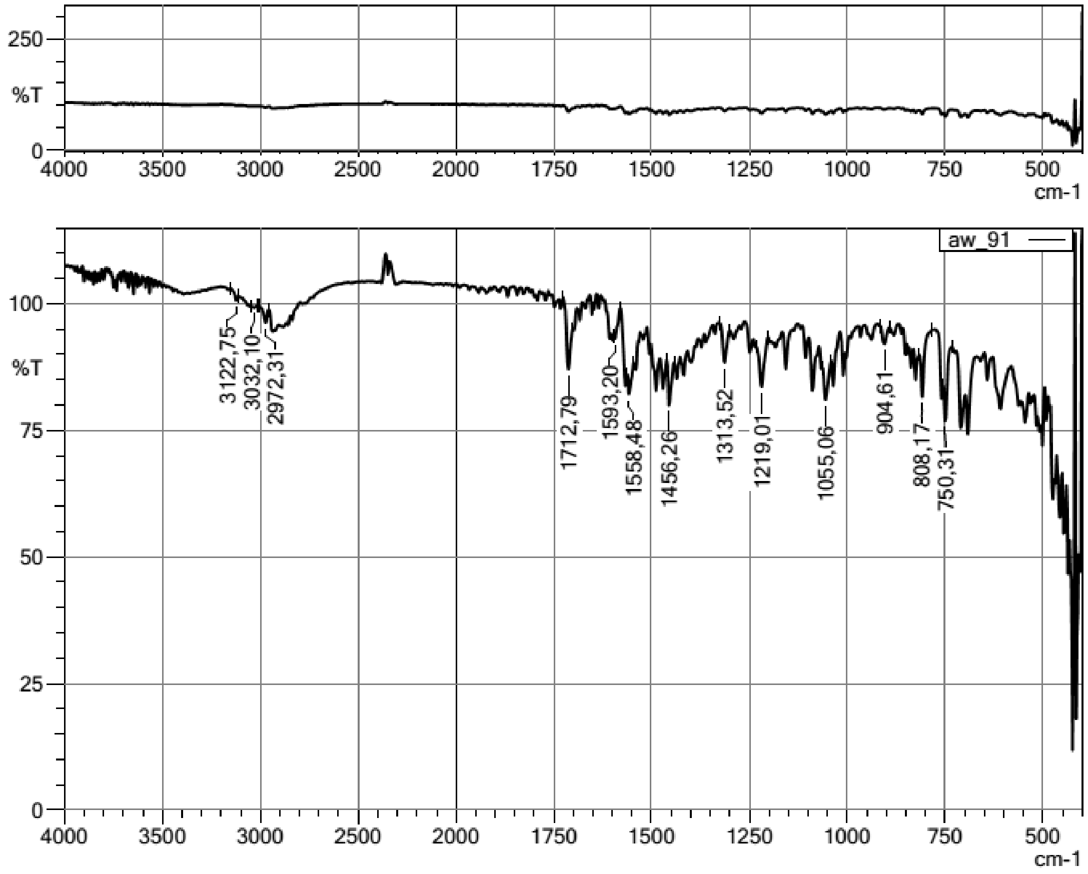

-HRMS spectrum of the compound 4g

Data File: C:\LabSolutions\Data\Analiz\Lyuttas\AW-9_10.lcd

Elmt	Val.	Min	Max	Elmt	Val.	Min	Max	Elmt	Val.	Min	Max	Elmt	Val.	Min	Max	Use Adduct
H	1	20	35	O	2	2	4	Cl	1	0	1	I	3	0	0	H
C	4	26	30	F	1	0	1	Br	1	0	1					
N	3	3	4	S	2	1	1	Ru	2	0	0					

Error Margin (ppm):	5	DBE Range:	not fixed	Electron Ions:	both
HC Ratio:	unlimited	Apply N Rule:	yes	Use MSn Info:	no
Max Isotopes:	3	Isotope RI (%):	1.00	Isotope Res:	10000
MSn Iso RI (%):	10.00	MSn Logic Mode:	AND	Max Results:	500

Event#: 1 MS(E+) Ret. Time : 7.960 -> 8.093 Scan# : 1195 -> 1215

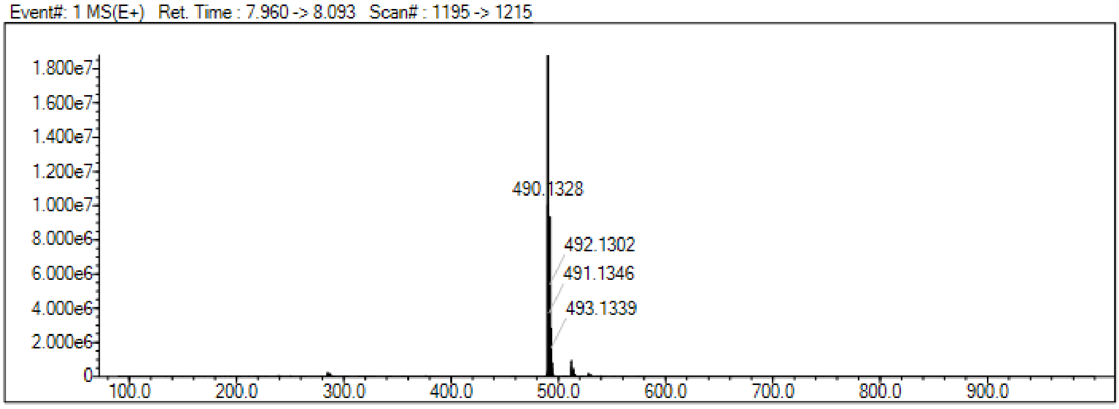

-^{1}H NMR spectrum of the compound 4g

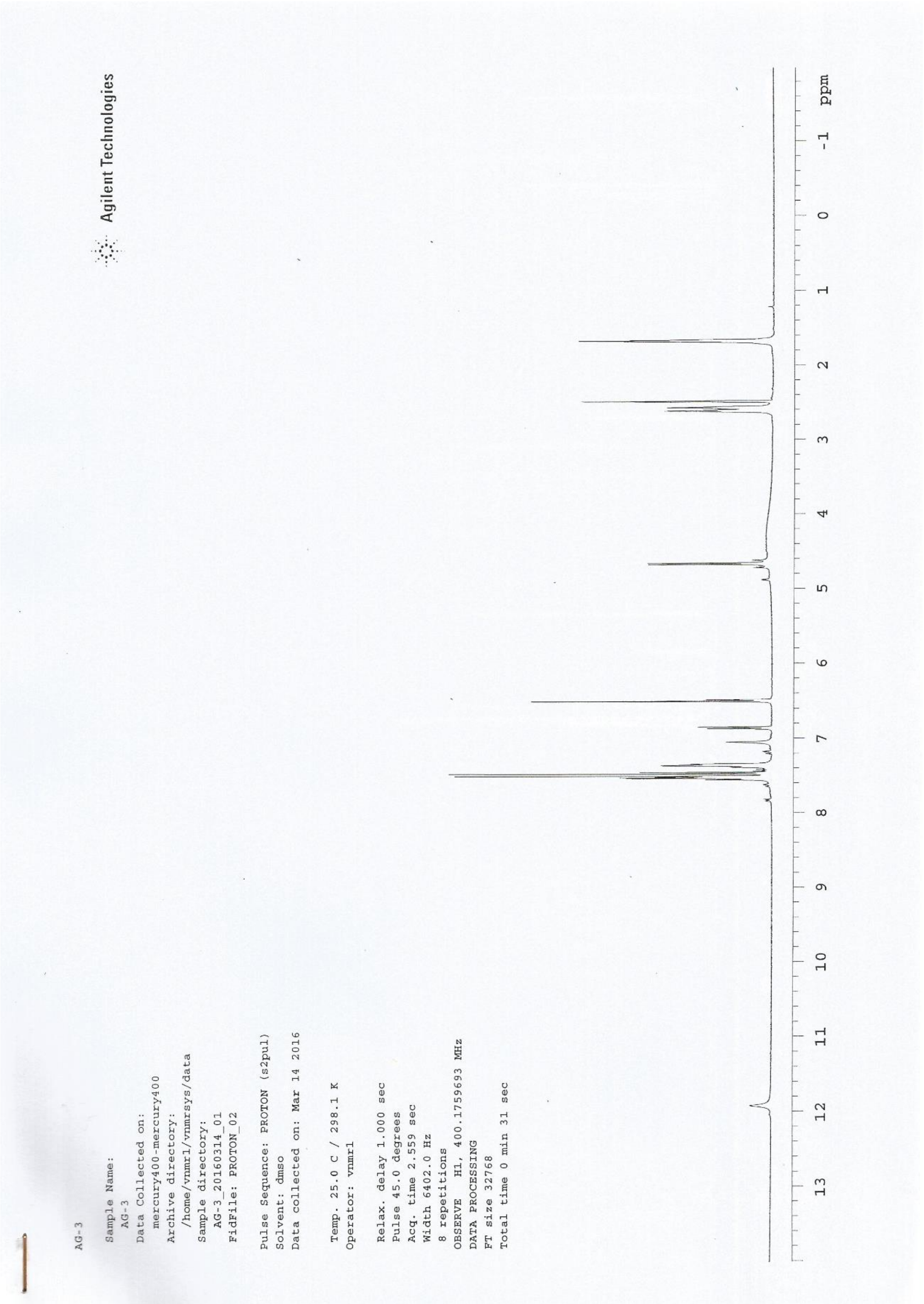

-^{13}C NMR spectrum of the compound 4g

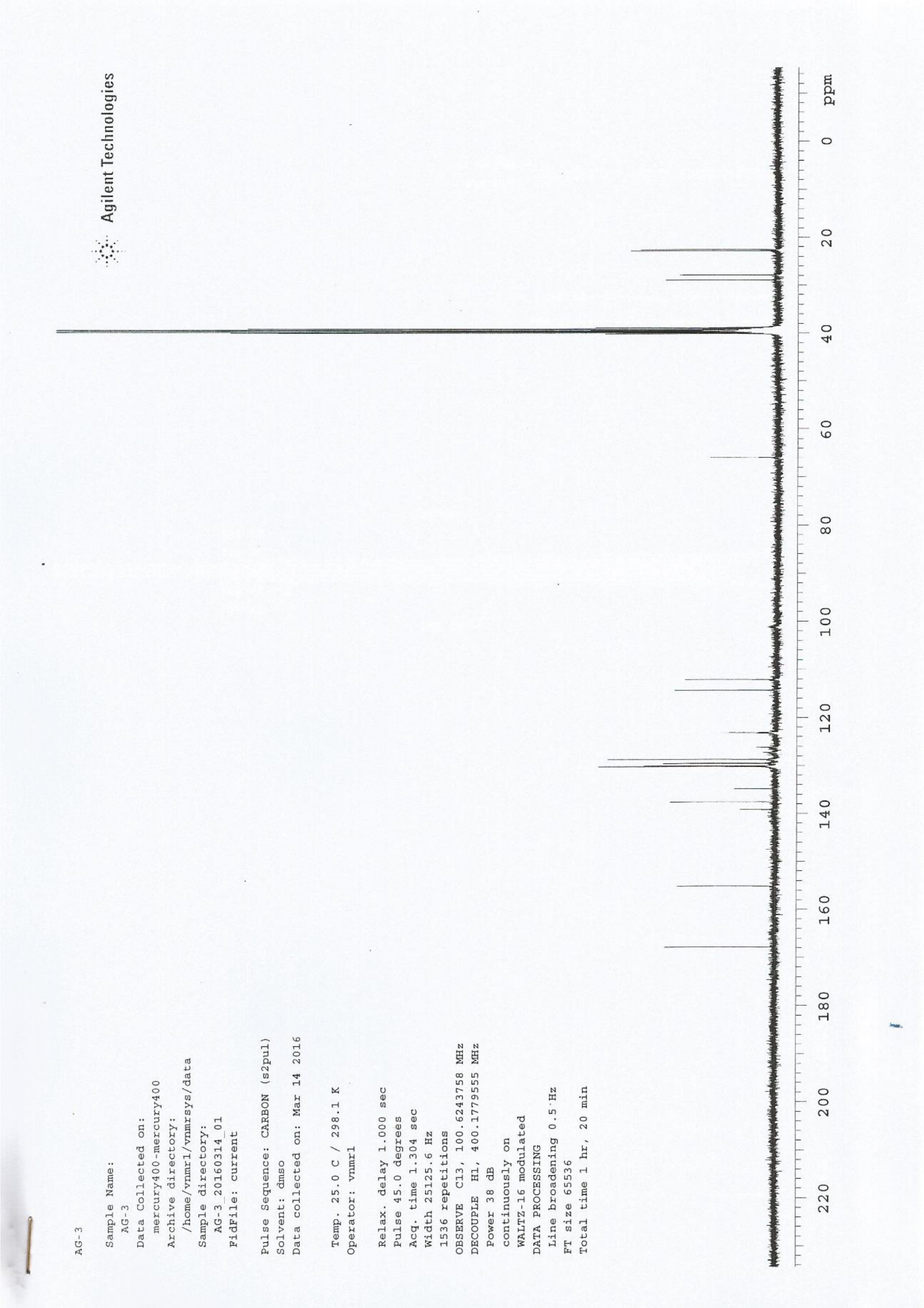

-IR spectrum of the compound 4h

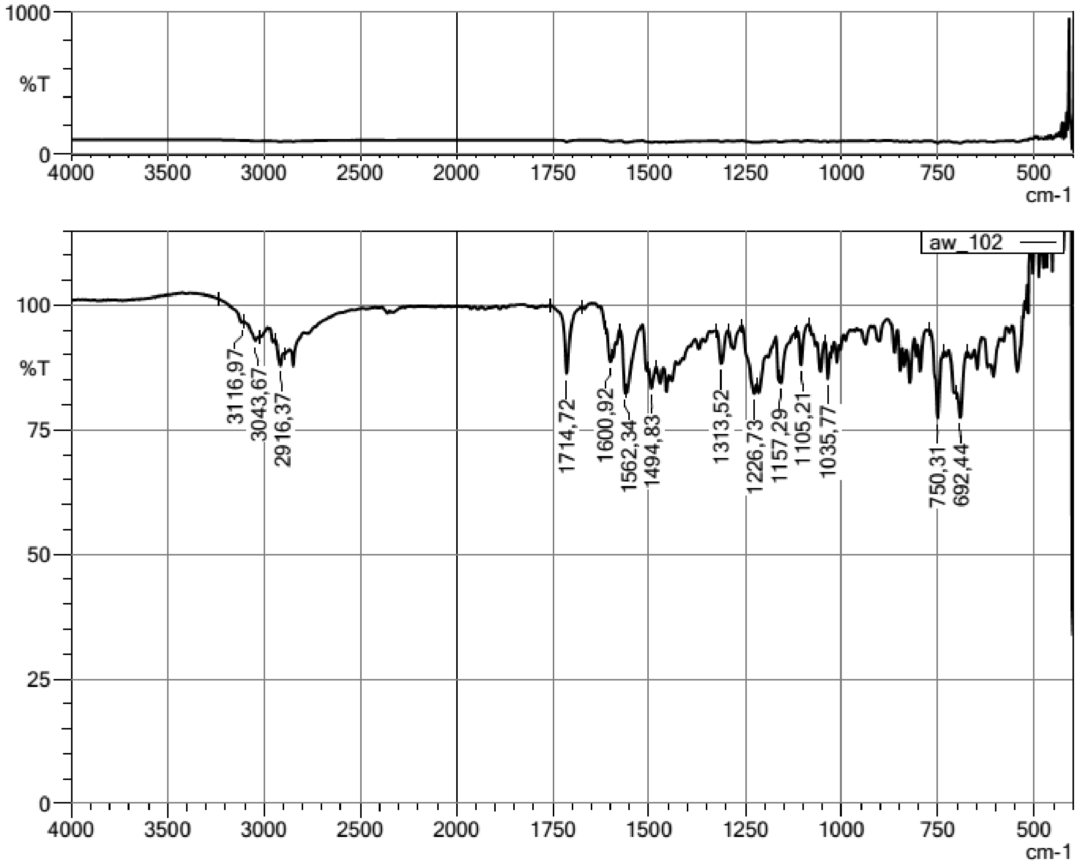

-HRMS spectrum of the compound 4h

Data File: C:\LabSolutions\Data\Analiz\Lyuttas\AW-10_13.lcd

Elmt	Val.	Min	Max	Elmt	Val.	Min	Max	Elmt	Val.	Min	Max	Elmt	Val.	Min	Max	Use Adduct
H	1	20	35	O	2	2	4	Cl	1	0	2	I	3	0	0	H
C	4	26	30	F	1	0	1	Br	1	0	1					
N	3	3	4	S	2	1	1	Ru	2	0	0					

Error Margin (ppm): 5
HC Ratio: unlimited
Max Isotopes: 3
MSn Iso RI (%): 10.00

DBE Range: not fixed
Apply N Rule: yes
Isotope RI (%): 1.00
MSn Logic Mode: AND

Electron Ions: both
Use MSn Info: no
Isotope Res: 10000
Max Results: 500

Event#: 1 MS(E+) Ret. Time : 7.480 -> 7.720 Scan# : 1123 -> 1159

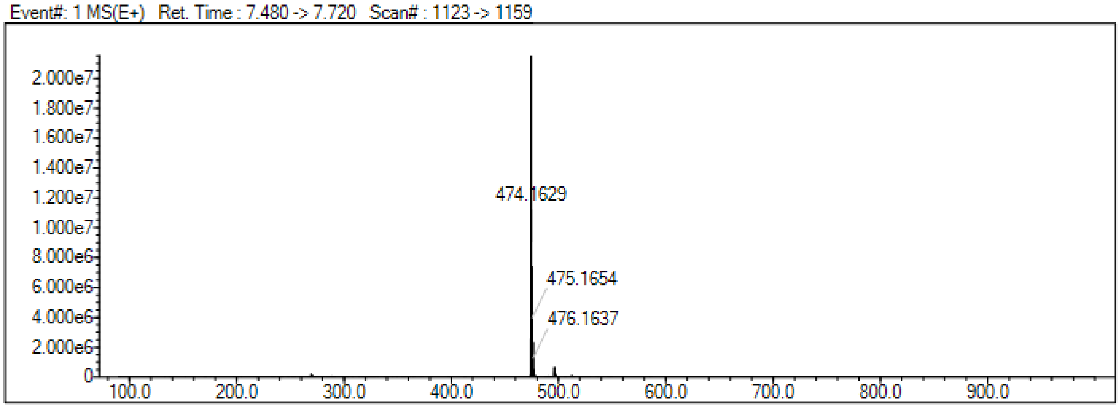

- ^{1}H NMR spectrum of the compound 4h

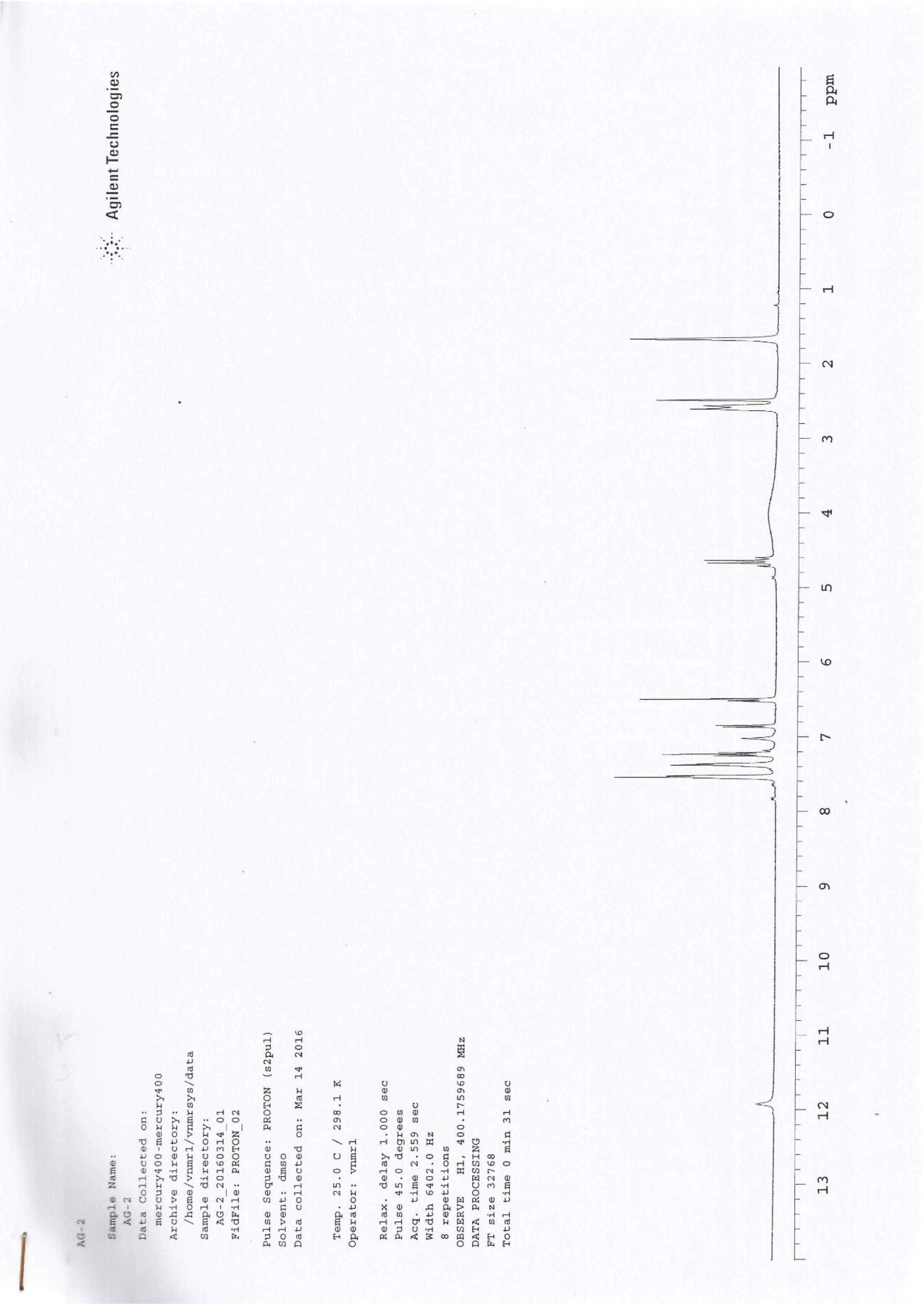

-^{13}C NMR spectrum of the compound 4h

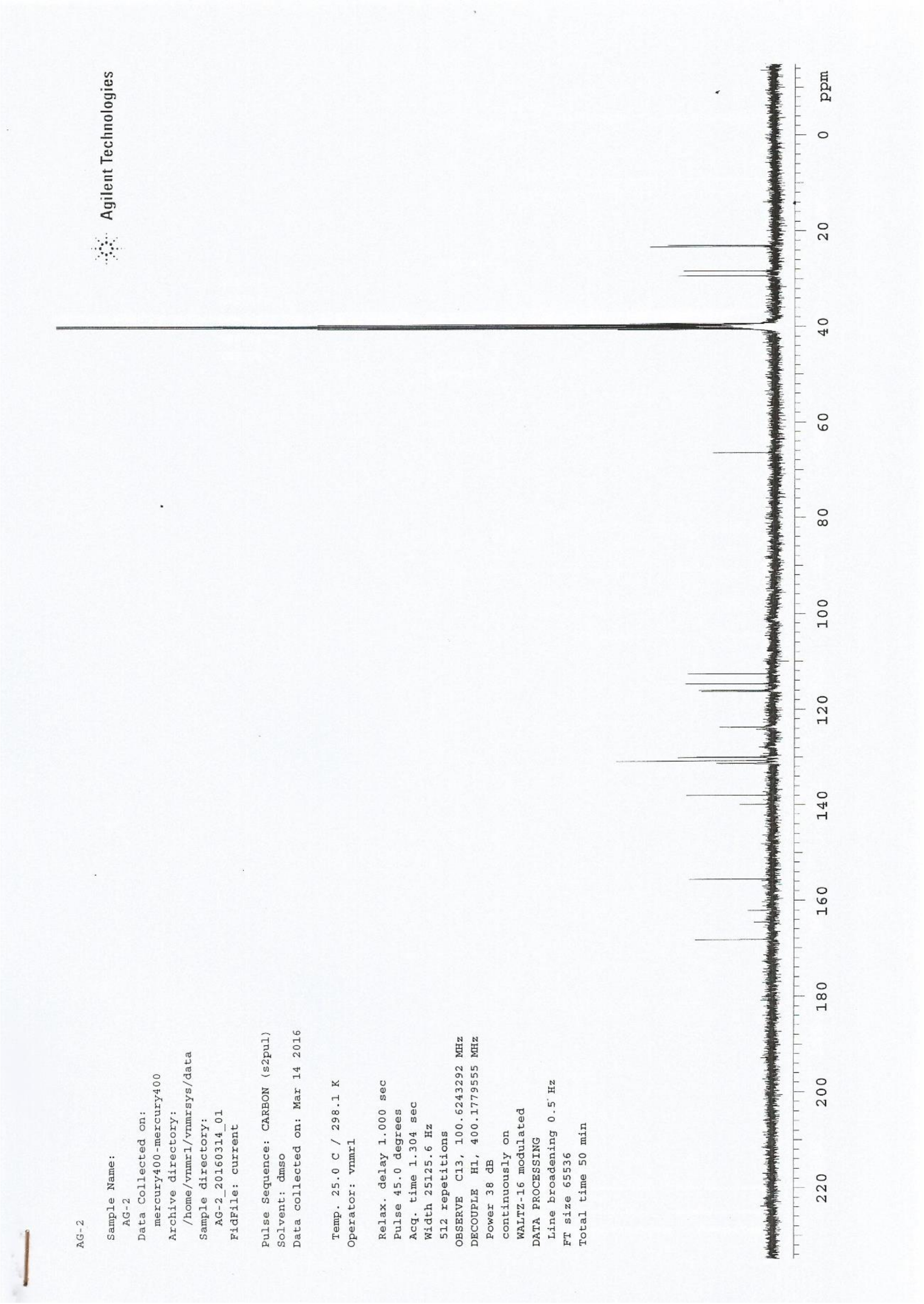

-IR spectrum of the compound 4i

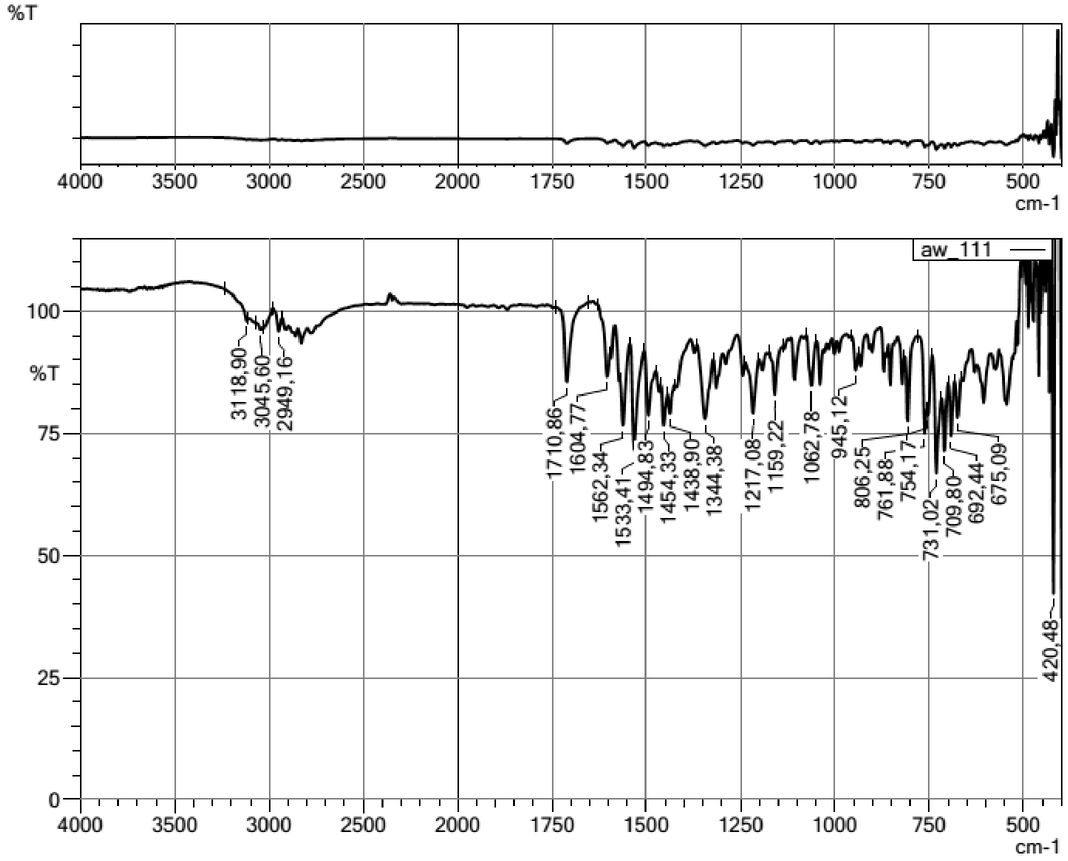

-HRMS spectrum of the compound 4i

Data File: C:\LabSolutions\Data\Analiz\Lyuttas\AW-11_10.lcd

Elmt	Val.	Min	Max	Elmt	Val.	Min	Max	Elmt	Val.	Min	Max	Elmt	Val.	Min	Max	Use Adduct
H	1	20	35	O	2	2	4	Cl	1	0	1	I	3	0	0	H
C	4	26	30	F	1	0	1	Br	1	0	1					
N	3	3	4	S	2	1	1	Ru	2	0	0					

Error Margin (ppm): 5
HC Ratio: unlimited
Max Isotopes: 3
MSn Iso RI (%): 10.00

DBE Range: not fixed
Apply N Rule: yes
Isotope RI (%): 1.00
MSn Logic Mode: AND

Electron Ions: both
Use MSn Info: no
Isotope Res: 10000
Max Results: 500

Event#: 1 MS(E+) Ret. Time : 7.640 -> 7.773 Scan# : 1147 -> 1167

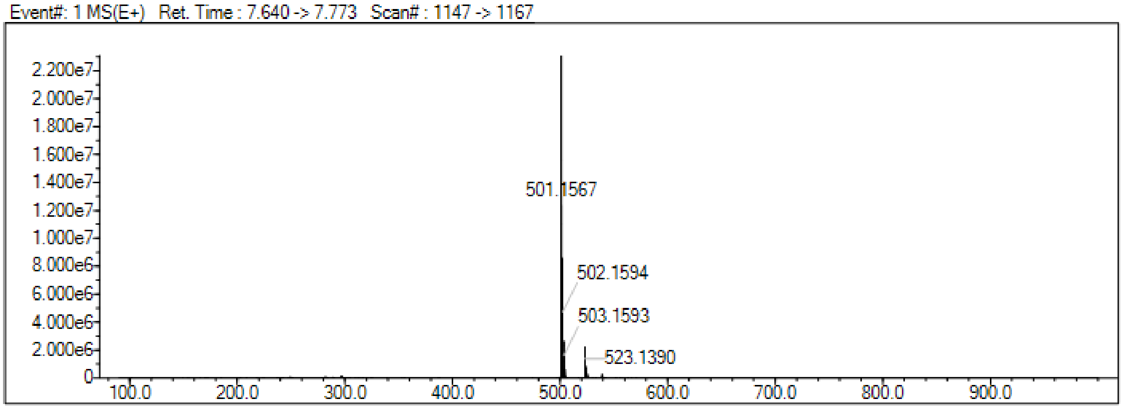

-^{1}H NMR spectrum of the compound 4i

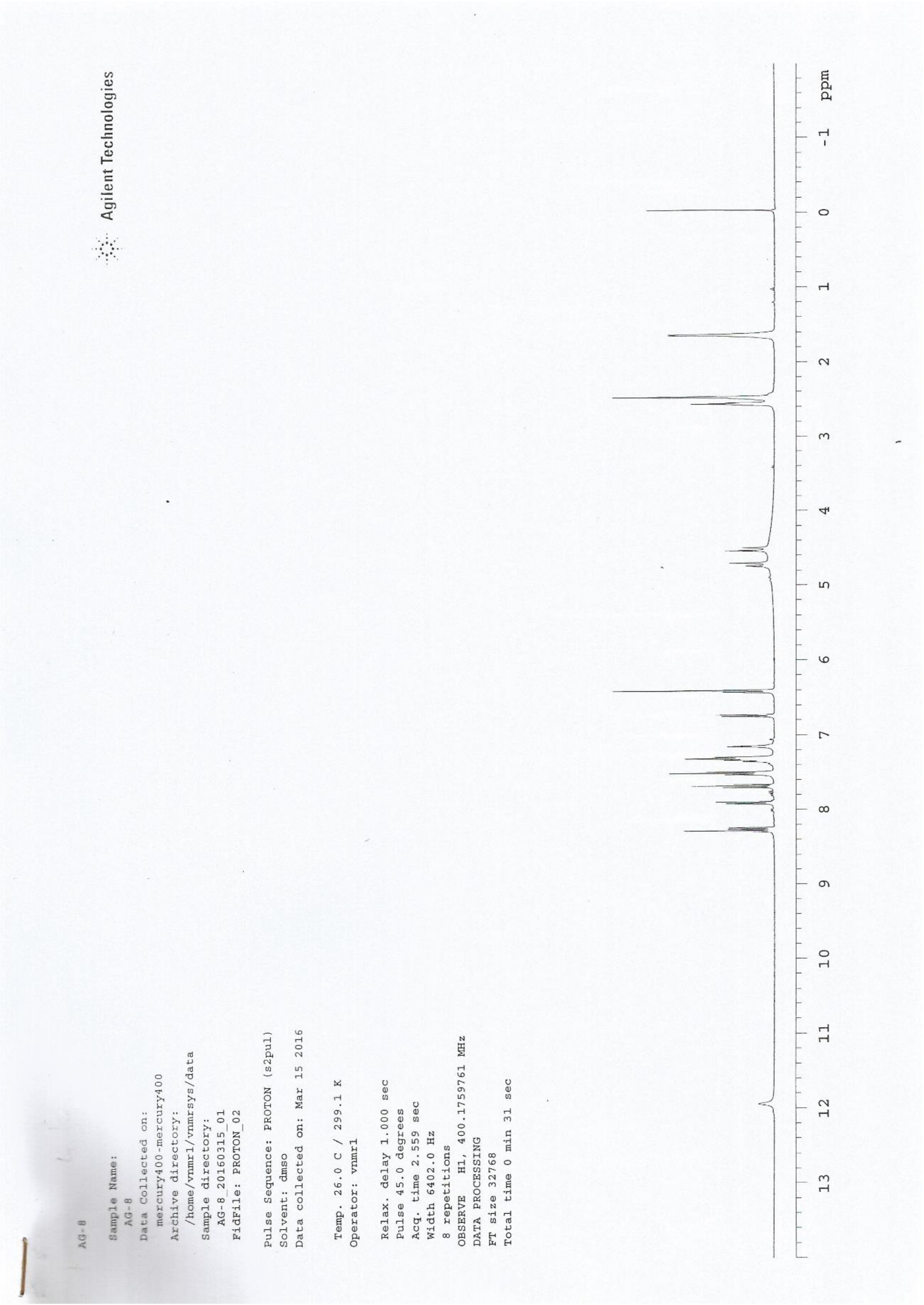

-^{13}C NMR spectrum of the compound 4i

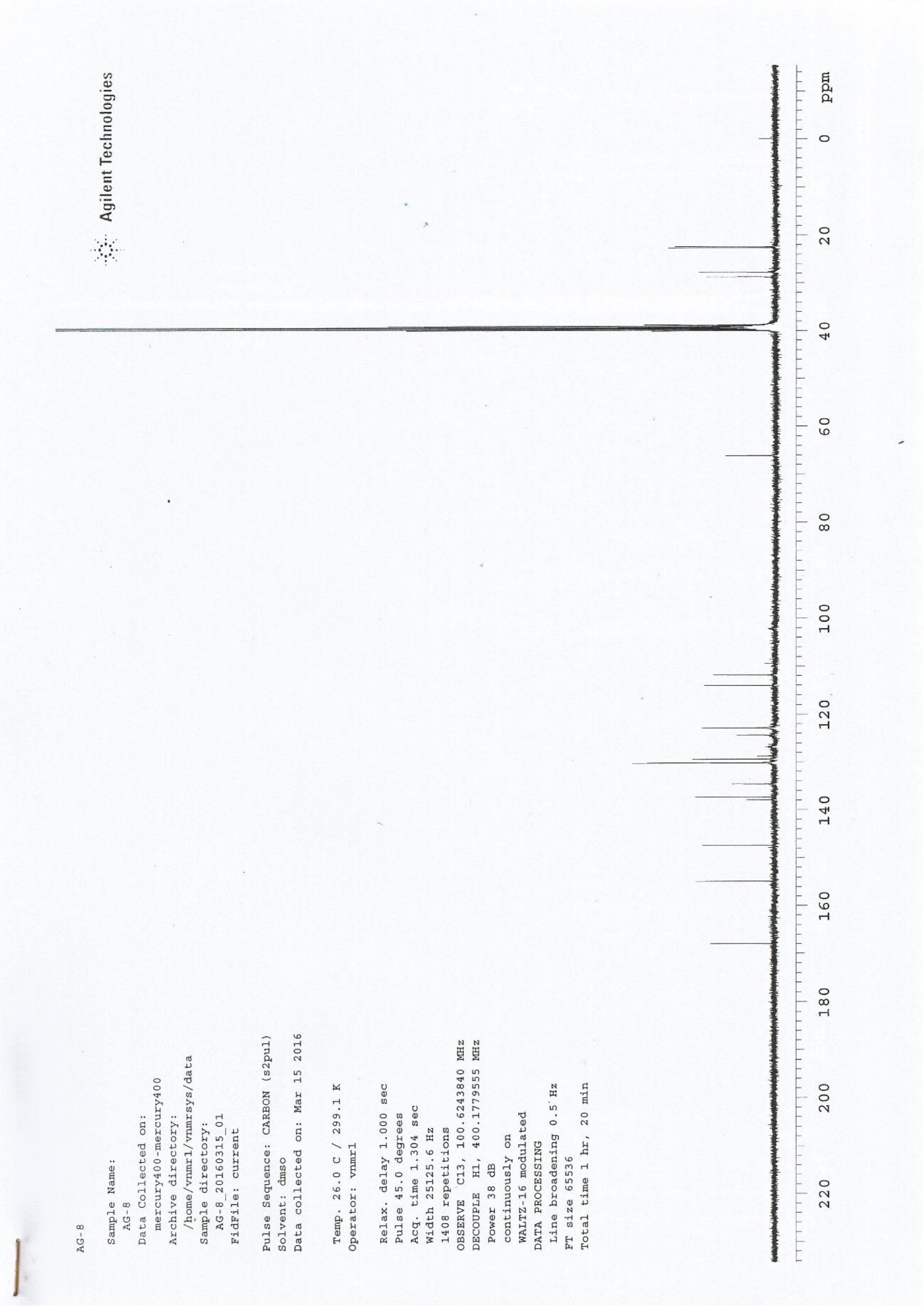

-IR spectrum of the compound 4j

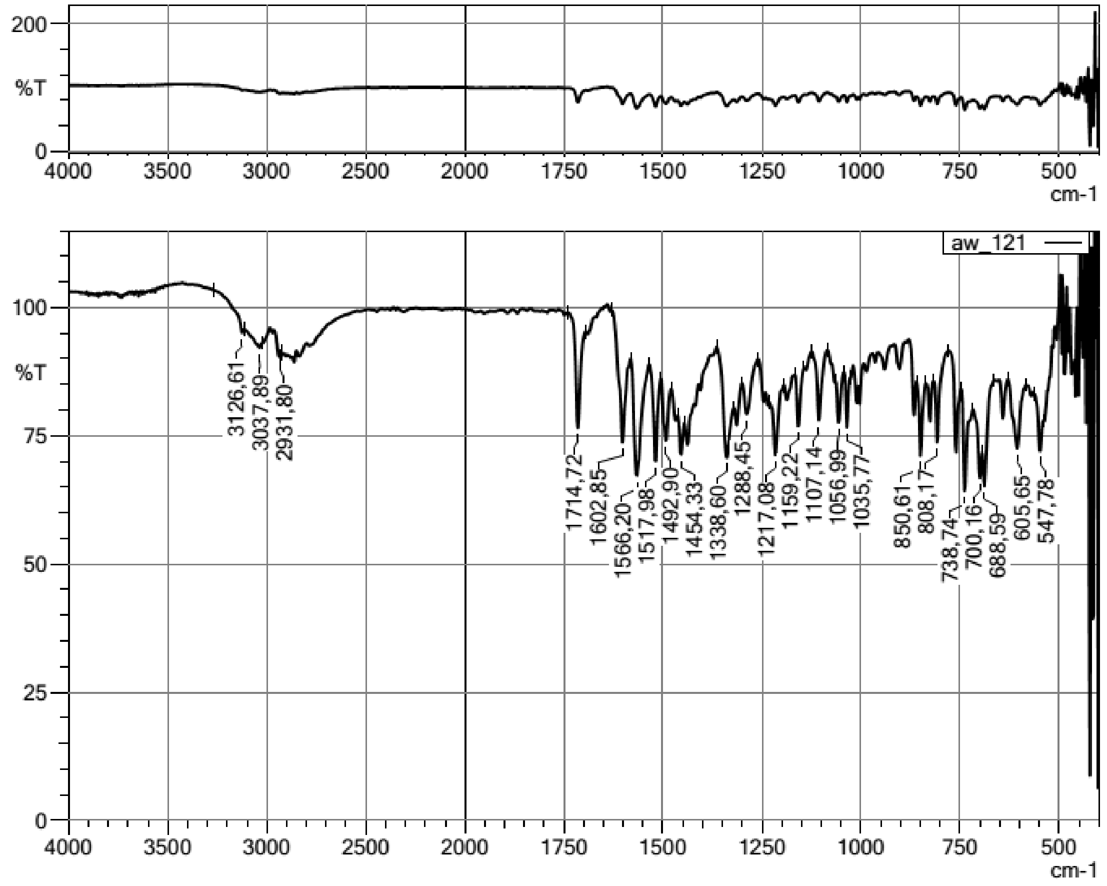

-HRMS spectrum of the compound 4j

Data File: C:\LabSolutions\Data\Analiz\Lyuttas\AW-12_11.lcd

Elmt	Val.	Min	Max	Elmt	Val.	Min	Max	Elmt	Val.	Min	Max	Elmt	Val.	Min	Max	Use Adduct
H	1	20	35	O	2	2	4	Cl	1	0	1	I	3	0	0	H
C	4	26	30	F	1	0	1	Br	1	0	1					
N	3	3	4	S	2	1	1	Ru	2	0	0					

Error Margin (ppm): 5
HC Ratio: unlimited
Max Isotopes: 3
MSn Iso RI (%): 10.00

DBE Range: not fixed
Apply N Rule: yes
Isotope RI (%): 1.00
MSn Logic Mode: AND

Electron Ions: both
Use MSn Info: no
Isotope Res: 10000
Max Results: 500

Event#: 1 MS(E+) Ret. Time : 7.707 -> 7.920 Scan# : 1157 -> 1189

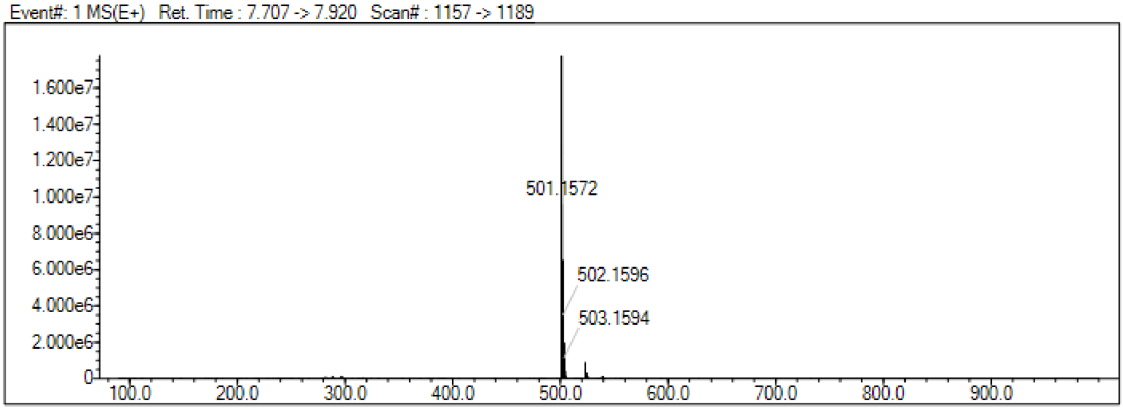

-¹H NMR spectrum of the compound 4j

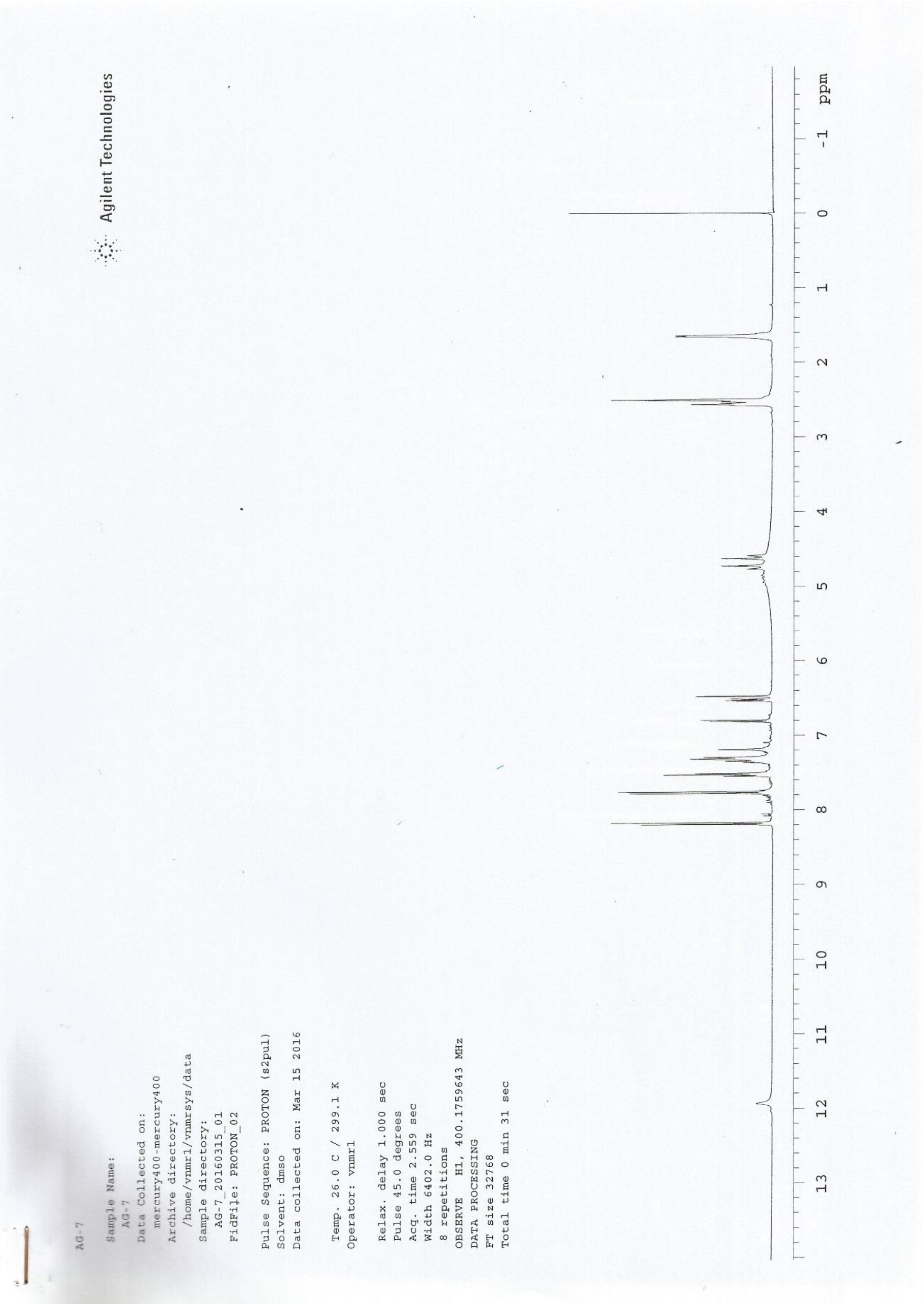

-^{13}C NMR spectrum of the compound 4j

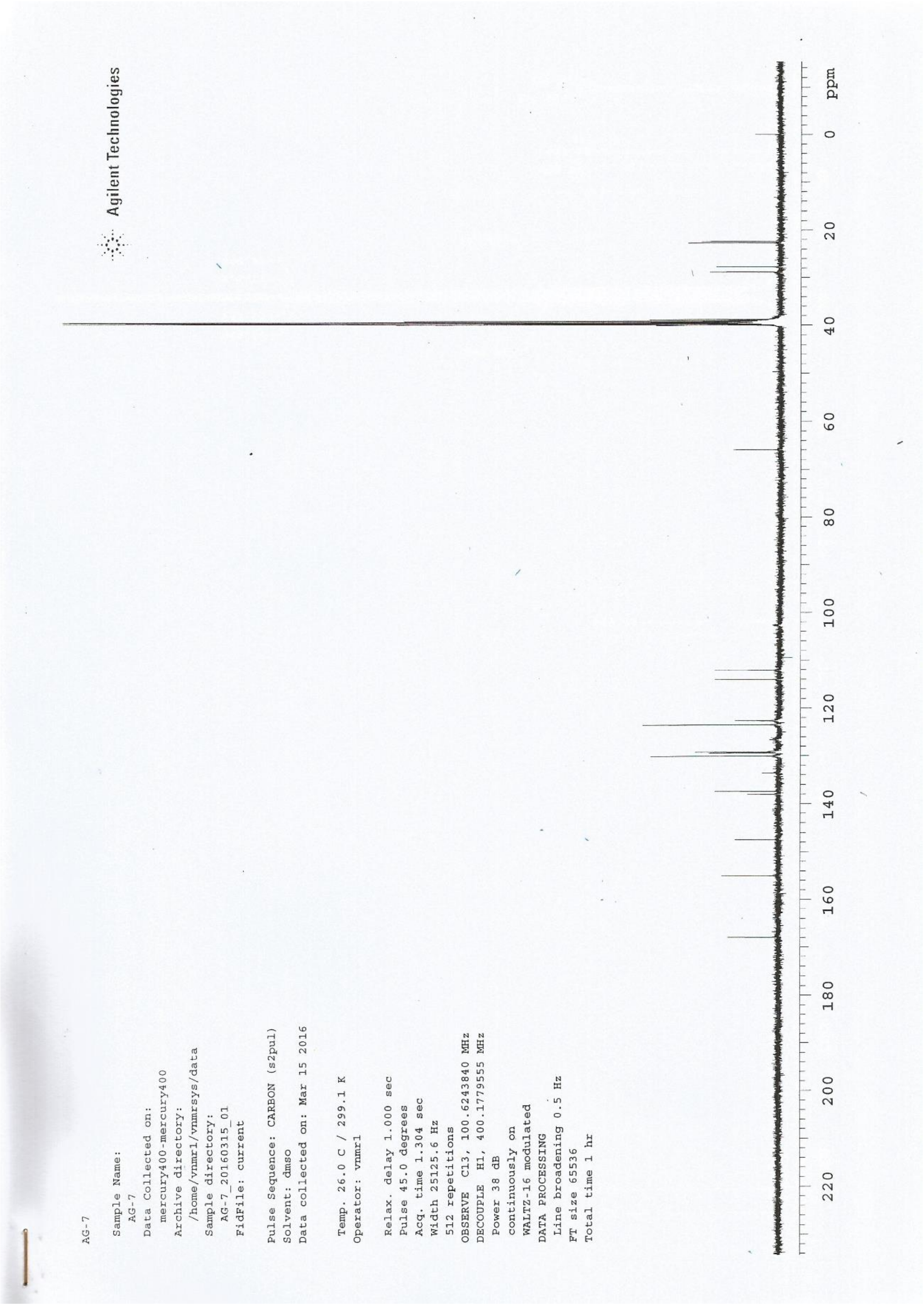

- IR spectrum of the compound 4k

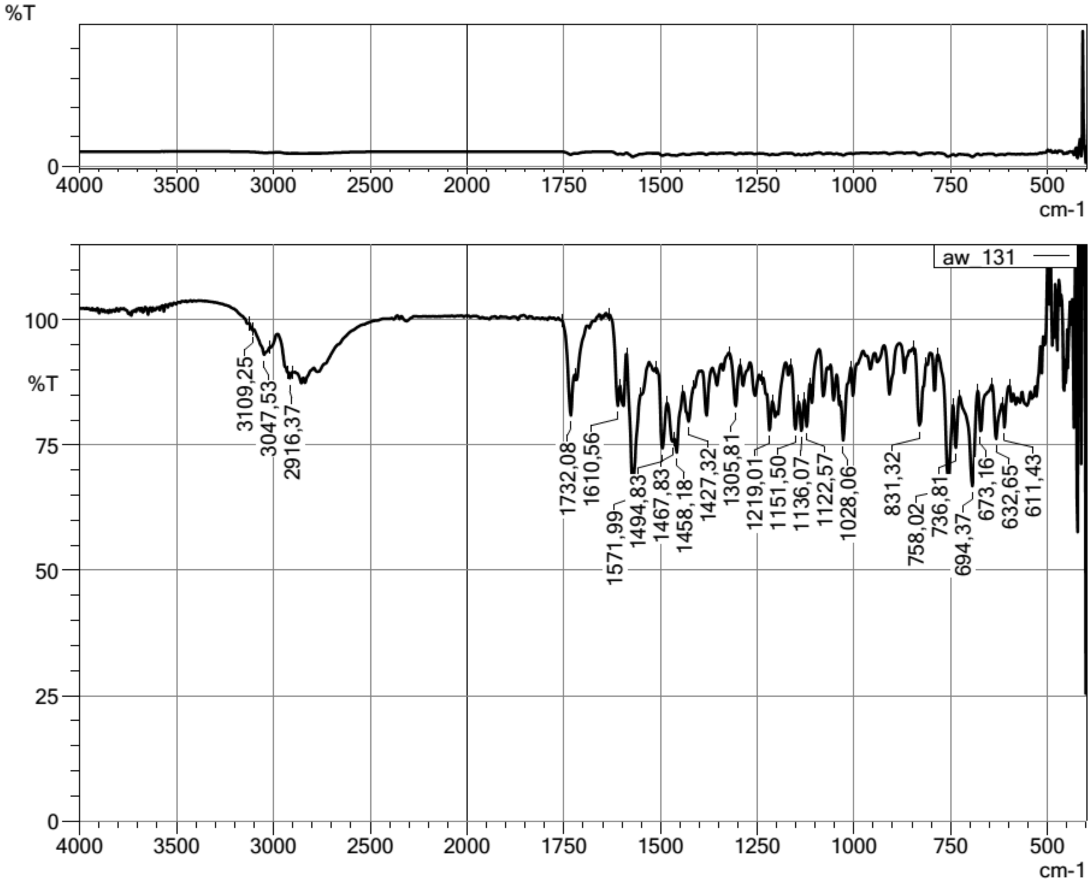

-HRMS spectrum of the compound 4k

Data File: C:\LabSolutions\Data\Analiz\Lyuttas\AW-13_12.lcd

Elmt	Val.	Min	Max	Elmt	Val.	Min	Max	Elmt	Val.	Min	Max	Elmt	Val.	Min	Max	Use Adduct
H	1	20	35	O	2	2	4	Cl	1	0	2	I	3	0	0	H
C	4	26	30	F	1	0	1	Br	1	0	1					
N	3	3	4	S	2	1	1	Ru	2	0	0					

Error Margin (ppm): 5
HC Ratio: unlimited
Max Isotopes: 3
MSn Iso RI (%): 10.00

DBE Range: not fixed
Apply N Rule: yes
Isotope RI (%): 1.00
MSn Logic Mode: AND

Electron Ions: both
Use MSn Info: no
Isotope Res: 10000
Max Results: 500

Event#: 1 MS(E+) Ret. Time : 8.840 Scan# : 1327

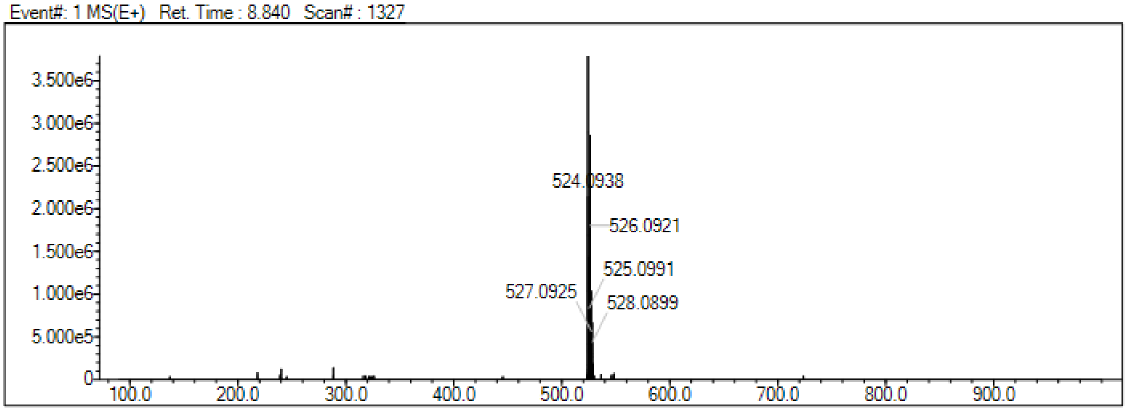

-^{1}H NMR spectrum of the compound 4k

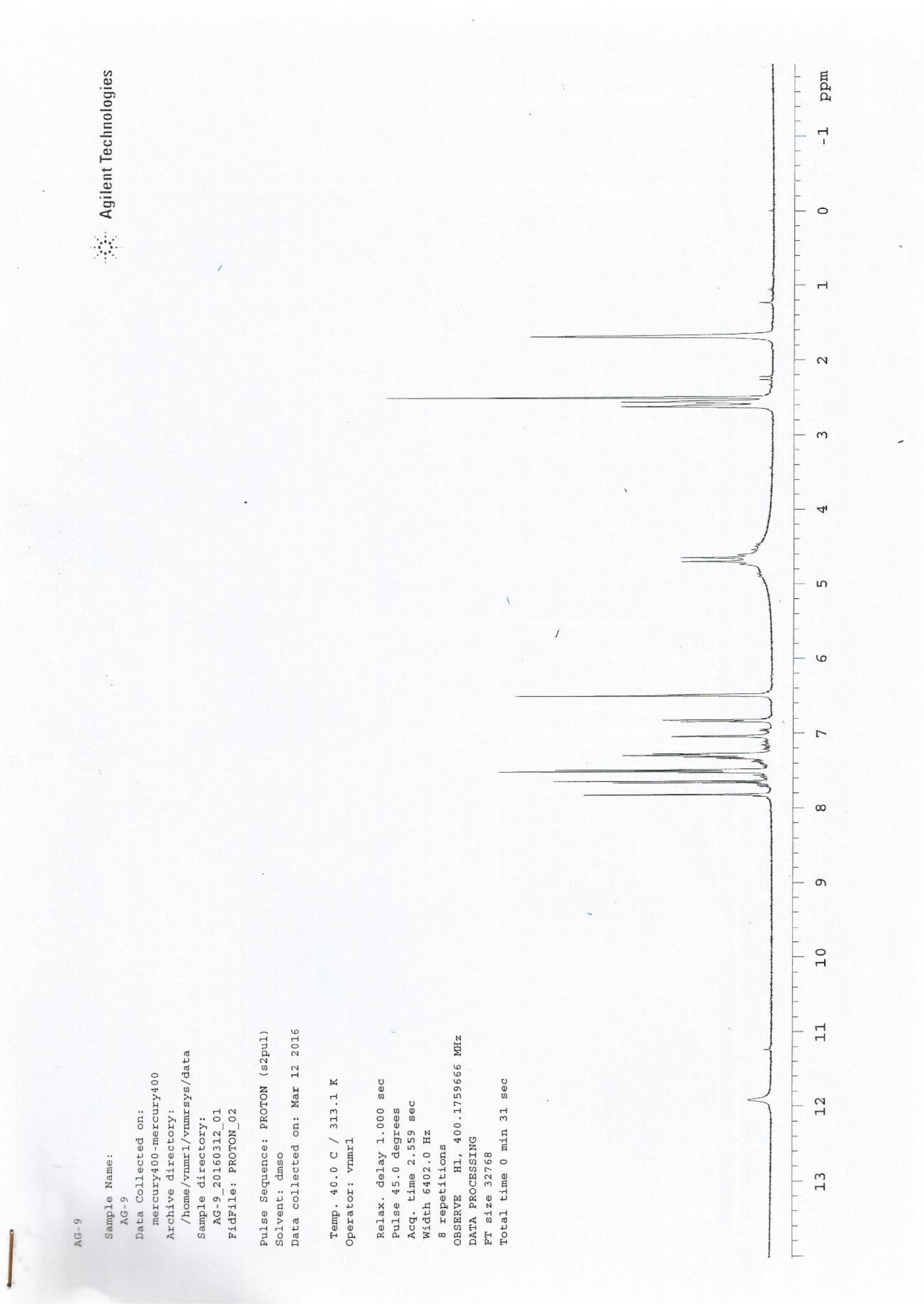

-^{13}C NMR spectrum of the compound4k

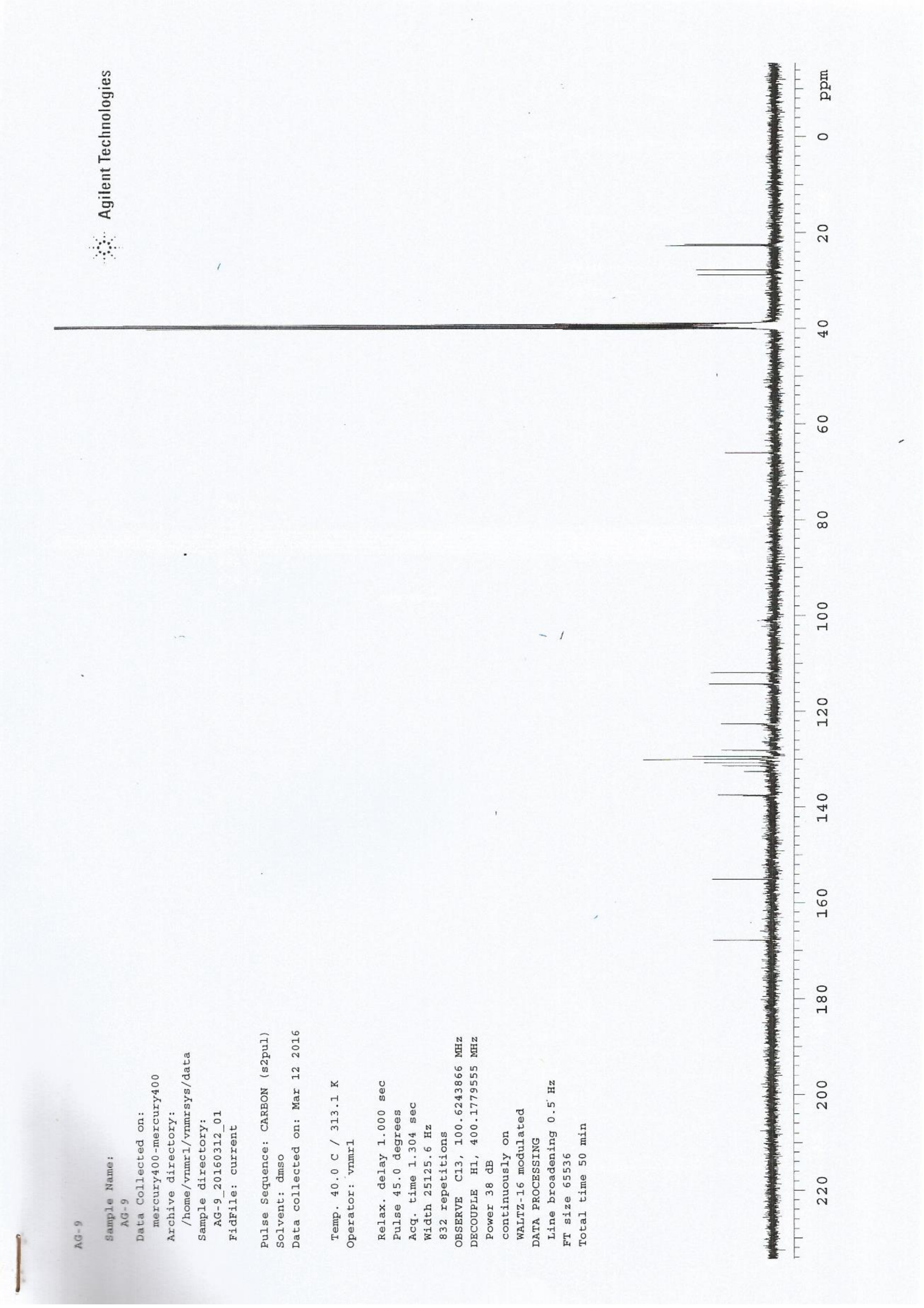

Supplement: Supplementary file 1 [file molecules-23-00135-s001.pdf]
